# Pharmacomodulation of G-quadruplexes in long non-coding RNAs dysregulated in colorectal cancer

**DOI:** 10.1186/s12915-025-02322-8

**Published:** 2025-08-08

**Authors:** Shubham Sharma, Jérémie Mitteaux, Angélique Pipier, Marc Pirrotta, Marie-José Penouilh, David Monchaud, Bhaskar Datta

**Affiliations:** 1 Department of Biological Sciences and Engineering, Indian Institute of Technology Gandhinagar, Gandhinagar, Gujarat 382055 India; 2 Institut de Chimie Moléculaire de l’Université de Bourgogne (ICMUB), Université Bourgogne Europe (UBE), CNRS UMR6302, Dijon, 21000 France; 3 Department of Chemistry, Indian Institute of Technology Gandhinagar, Gandhinagar, Gujarat 382055 India

**Keywords:** Colorectal cancer, Long non-coding RNA, G-quadruplex, Ligands, Molecular helicases

## Abstract

**Background:**

Non-coding RNAs (ncRNAs) in human cells constitute a substantial portion of the transcriptome but do not lead to protein synthesis. Among them, long non-coding RNAs (lncRNAs, > 200 nucleotides long) are fascinating in their ability to orchestrate critical cellular functions that govern cell development, differentiation, and metabolism. Therefore, the dysregulation of lncRNAs has been linked with several diseases, chiefly cancers.

**Results:**

We focused here on colorectal cancer (CRC), the second-highest cause of mortalities related to cancer worldwide, and more particularly on three lncRNAs, *i.e.*, *LINC01589*, *MELTF-AS1*, and *UXT-AS1*, known to be dysregulated in CRC. We identified a vulnerability in these lncRNAs that could be exploited from a therapeutic point of view: a part of their sequence folds into a secondary structure referred to as G-quadruplex (G4), which is suspected to play active roles in the lncRNA functions. We demonstrate here that these sequences do fold into G4s both in vitro and in CRC cells, and that these G4s can be modulated using PhpC, a prototype molecule for destabilizing G4s.

**Conclusion:**

We describe an innovative anticancer strategy that fully abides by the rules of chemical biology. We indeed modulate the formation of G4s in cells using ad hoc molecular tools in the aim of disturbing the homeostasis and inner functioning of lncRNAs. By exploiting cellular outcomes, we infer how this pharmacomodulation affects CRC biology and, beyond this, the fate of CRC cells owing to the flawed repertoire of correction and/or compensatory mechanisms in cancer cells.

**Supplementary Information:**

The online version contains supplementary material available at 10.1186/s12915-025-02322-8.

## Background

Long non-coding RNAs (lncRNAs) represent an important class of ncRNAs within the human transcriptome, encompassing pivotal functions in both normal cellular physiology and disease pathogenesis [1, 2]. These RNAs are characterized by their length (> 200 nucleotides), translationally silent nature (with a few exceptions), and tissue-specific expression patterns. Their unique and intricate three-dimensional structures, in conjunction with finely orchestrated spatiotemporal expression patterns, make lncRNAs key players in the regulation of cell growth, differentiation, and developmental processes, acting at both genetic and epigenetic levels [3–6]. These regulatory roles also explain why lncRNAs are often dysregulated in various pathologies, including cancers [7–10].

**Fig. 1:**
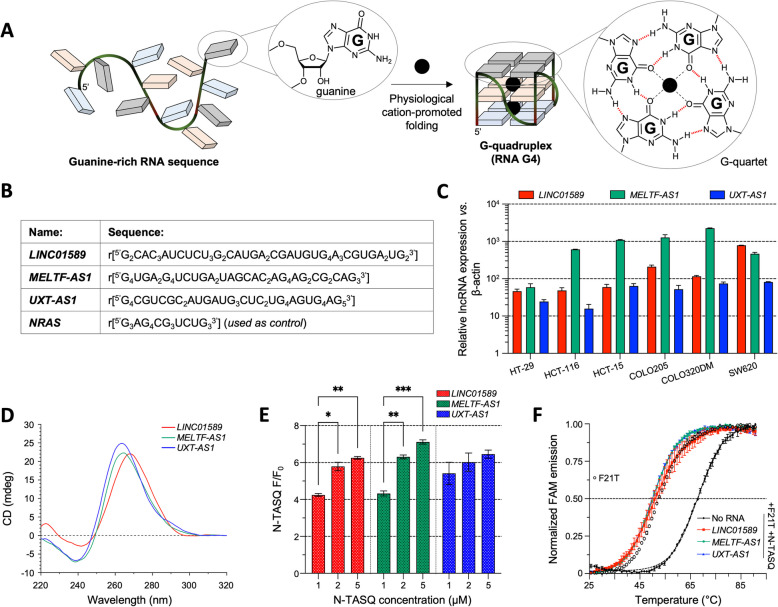
**A** Schematic representation of the folding of a G-quadruplex (G4) from a guanine (G)-rich RNA sequence. **B** Selected putative G4-forming sequences (PQSs) in *LINC01589*, *MELTF-AS1*, and *UXT-AS1* lncRNAs along with the sequence of *NRAS*, used as a positive RNA G4 control herein (sequences from 5' to 3')*.* **C** Reverse transcription-quantitative polymerase chain reaction (RT-qPCR) analyses performed with RNAs isolated from different CRC cell lines (HT-29, HCT-116, HCT-15, COLO205, COLO320DM, and SW620) to assess the relative *LINC01589*, *MELTF-AS1*, and *UXT-AS1* expression with respect to β-Actin (2^−△Ct^) ± SD. **D** CD spectroscopy performed with folded synthetic RNAs (1 µM). **E** N-TASQ fluorescence enhancement assay performed with folded synthetic RNAs (2 µM) against increasing concentrations of N-TASQ (1 to 5 µM). **F** Förster Resonance Energy Transfer-Melting Competition (FRET-MC) assay performed with folded F21T (0.2 µM) in the presence of N-TASQ (1 µM) and an excess of folded synthetic RNAs (3 µM). The results are collected from triplicates (*n* = 3) across three independent studies (*n* = 3). Panels **F** and **D**: a representative, unique experiment (**D**) or technical triplicate (**F**) is represented, for the sake of clarity. Two-way ANOVA was employed for the statistical analyses with *: *P* ≤ 0.05, **: *P* ≤ 0.01, ***: *P* ≤ 0.001; non-significant *P*-values are not represented

Among the structural motifs lncRNAs are replete with, G-quadruplexes (G4s) rank high. G4s are thermodynamically stable secondary structures that fold from guanine (G)-rich sequences owing to the ability of Gs to self-associate to form G-quartets, which then self-stack to form the G4 (Fig. 1A) [11–18]. The ncRNA G4s are involved in several ncRNA-related processes, from the docking of RNA binding proteins (RBPs) to miRNA maturation, from the control of the ncRNA stability to that of its cellular localization. While *in silico* transcriptome-wide analyses showed the high G4 content of lncRNAs [19], the demonstration of their existence in cells was challenging, owing to their dynamic regulation by chaperones and helicases, which make G4 formation transient [15, 20]. The flagship examples of G4-containing lncRNAs are undoubtedly *NEAT1* and *MALAT1*, both of which have been detected during transcriptome-wide profiling in vitro by the rG4-seq method [21] and in vivo by the G4RP-seq method [20, 22]. While the biological functions of *NEAT1* and *MALAT1* are well-known [23], the roles that their G4s play remain to be fully understood: a conventional and global approach to do this is to use G4-interacting compounds and exploit the cellular outcomes of their incubation for mechanistic interpretations. For instance, TMPyP4 [24] and PDS [25] were used to disrupt the interaction of *NEAT1* [26] and *MALAT1* G4s [27], respectively, with their protein partners, in both cases here NONO (for non-POU domain-containing octamer binding protein), known to play key roles in gene regulation and DNA repair [28]. It is intriguing that the same goal (impairing G4/NONO interaction) is achieved through two opposite strategies, as TMPyP4 is used to distort G4 (thus modifying the protein binding site) while PDS is used to stabilize it (thus precluding the access of the protein). The strategy we propose herein aims at benefitting from the advantages of these two approaches, without suffering from their limitations: on one hand, even if TMPyP4 is documented as a possible G4-unfolder [26, 29–32], its well-established G4-stabilizing properties [33, 34] blur the relevance of the first approach; on the other hand, even if PDS is used to target RNA G4s [35–37], its well-established DNA-interacting properties [38–41], which strongly jeopardizes genetic stability, reduce the relevance of the second approach. That is why we decided herein to further investigate the relevance of the first strategy which allows for harnessing the roles of lncRNA G4s without triggering genetic instability, using a more optimized molecular tool (*vide infra*). To better imbue this strategy with therapeutic value, we also decided to focus on G4s that may play regulatory roles in lncRNAs found to be dysregulated in colorectal cancer (CRC). This is justified because CRC is the second-highest cause of mortalities related to cancer worldwide, with > 900,000 deaths each year. While recent improvements in therapies and surgery have resulted in a 5-year survival rate of ~ 64%, the molecular basis of CRC progression remains unclear at present [42, 43]. LncRNAs play a prominent role in CRC progression and their dysregulation and/or mutations have been studied for both fundamental (tumorigenesis or metastasis) and applied research (diagnosis and prognosis) [44, 45]. In spite of these appealing characteristics, the dysregulation of lncRNAs in CRC is less explored compared to other RNAs, and the questions pertaining to the roles that G4s may have in their mechanism of action remain to be further investigated [44–46]. It has been recently shown that G4s in CRC-dysregulated lncRNAs *GSEC*, *REG1CP*, and *LUCAT1* might be involved in CRC pathogenesis [47–49]; however, their role in the regulation of other lncRNAs known to be dysregulated in CRC such as *LINC01589* (*CTA-941F9.9*), *MELTF-AS1* (*MFI2-AS1*), and *UXT-AS1* [50–52], with implications in cell proliferation, migration, and apoptosis, remain to be understood and pharmacologically exploited, which is precisely the aim of our current investigations.

In this work, following a thorough in silico assessment of G4 content in lncRNAs dysregulated in CRC, we first assessed the expression of *LINC01589*, *MELTF-AS1*, and *UXT-AS1* in a series of CRC cells and then showed that G4s can fold within these lncRNAs both in vitro (*via* a series of established techniques including circular dichroism (CD), thermal difference spectra (TDS), fluorescence titrations, melting assays (FRET-MC) and mass spectrometry) and in CRC cells (via the G4-RNA-specific precipitation (G4RP) technique) [22, 53–55]. Next, we demonstrated that these G4s are targetable in CRC cells, ironing them out using PhpC, a molecule currently under study for its G4-destabilizing properties [56–58], which represents an unprecedented strategy to fight against the cancer-specific upregulation of these G4-containing lncRNAs. Our results thus demonstrate the potential of these lncRNA G4s to be used as targets for therapeutic interventions.

## Results and discussion

### The G4 content of lncRNAs dysregulated in CRC

We started our investigations by assessing the putative G-quadruplex-forming sequence (PQS) content of lncRNAs dysregulated in CRC (Additional file 1: Fig. S1). The list of these lncRNAs was obtained from Lnc2Cancer 3.0, and the corresponding FASTA sequences were retrieved from the NCBI Nucleotide database [59, 60]. Out of 952 entries, only 611 (corresponding to 260 unique lncRNAs) had validated or reviewed RefSeq sequences. The PQS content of these sequences was analyzed using QGRS mapper (max length: 45; min G-group: 2; loop size: 0 to 36) [61]: most of these lncRNAs (257 out of 260 lncRNAs, 99%) had at least one PQS bearing two G-quartets (2G-PQS, 99%), 120 had at least one PQS bearing three G-quartets (3G-PQS, 46%) and 8 had at least one PQS bearing four G-quartets (4G-PQS, 3%). We also evaluated the PQS content using G4Hunter (window size: 45; threshold: 0.9) [62, 63]: 134 had at least one 2G-PQS (51%), 101 had at least one 3G-PQS (39%) and 7 had at least one 4G-PQS (3%). Quite satisfyingly, *LINC01589* (NR_131244.1, 593-nt), *MELTF-AS1* (NR_038285.1, 951-nt), and *UXT-AS1* (NR_028119.1, 565-nt) were found to be highly G4-rich. The high-scoring 2G-, 3G-, and 4G-PQSs within *LINC01589*, *MELTF-AS1*, and *UXT-AS1*, respectively, were selected for subsequent experiments (Fig. 1B, Additional file 1: Fig. S1; Additional file 2: Table S1).

### Expression of *LINC01589*, *MELTF-AS1*, and *UXT-AS1* lncRNAs in CRC

We next assessed the expression levels of *LINC01589*, *MELTF-AS1*, and *UXT-AS1* lncRNAs in different CRC cells (also in different cancer cell lines, Additional file 1: Fig. S2). RT-qPCR analyses (Additional file 3: Table S2) were performed using RNA isolated from CRC cells, *i.e.*, HT-29, HCT-116, HCT-15, COLO205, COLO320DM, and SW620: the relative lncRNA expression (with respect to β-actin) ranged from 46.4 to 784.1 for *LINC01589* (lowest: HT-29, highest: SW620); 59.3 to 2251.8 for *MELTF-AS1* (lowest: HT-29, highest: COLO320DM); 15.8 to 81.7 for *UXT-AS1* (lowest: HCT-116, highest: SW620) (Fig. 1C). These results showed the cell line-dependent variations in the level of *LINC01589*, *MELTF-AS1*, and *UXT-AS1* lncRNAs but also confirmed that they are *bona fide* good targets for assessing the existence of G4s in CRC-dysregulated lncRNAs.

### *LINC01589*, *MELTF-AS1*, and *UXT-AS1* lncRNAs form stable G4s in vitro

Several techniques were then implemented to confirm the formation of G4s in the above-mentioned PQSs selected within *LINC01589* (45-nt), *MELTF-AS1* (41-nt), and *UXT-AS1* (41-nt) lncRNAs in vitro (Fig. 1B; Additional file 4: Table S3). For simplicity, these PQSs are referred to hereafter by the name of their cognate lncRNAs. CD spectroscopy [64] indicated that the signatures obtained for these lncRNAs could be compatible with a parallel G4 fold (maxima at *ca*. 265 and minima at *ca*. 240 nm, Fig. 1D) [18] but this rather global analysis must be confirmed by complementary techniques. To this end, we performed TDS investigations [65] and the signatures obtained (Additional file 1: Fig. S3) confirmed unambiguously the G4 fold of *MELTF-AS1* and *UXT-AS1*, while that of *LINC01589* might indicate a possible mixture of structures. To go a step further, fluorescence titrations were performed using NaphthoTASQ, or N-TASQ (Fig. 1E, Additional file 1: Fig. S4). N-TASQ is a biomimetic ligand that assembles into a template-assembled synthetic G-quartet, or TASQ [66, 67], its template being a fluorogenic naphthalene. N-TASQ is also a twice-as-smart G4 ligand, being both a smart ligand (whose conformation is modified upon interaction with the G4 targets) and a smart fluorescent probe (whose fluorescence is switched on upon interaction with the G4 targets) [68]. It has been used both in live and fixed cells, notably for assessing the modulation of G4 landscapes upon incubation with G4-interacting molecules such as BRACO-19, TMPyP4 [69] and PhpC (*vide infra*) [57], but also for discriminating between different tumor types (ALT *versus* telomerase-positive cancers) [70]. Its turn-on fluorescence properties originate in a disruption of the electronic communication between the TASQ template and its guanine arms (which restores the fluorescence of the naphthalene template), triggered by the conformational switch that the probe experiences when binding to G4s [71, 72]. All TASQs interact with G4s via their intramolecular synthetic G-quartet [67], which makes them pan-G4 ligands; they interact with DNA and RNA G4s with a similar affinity with an apparent affinity constant (^app^*K*_D_) lies between 0.5 and 1.0 mM [57, 73], determined by fluorescence quenching assay (FQA; Additional file 1: Fig. S5) [74]. We further showed here that it interacts efficiently with *LINC01589*, *MELTF-AS1*, and *UXT-AS1* G4s in vitro by both CD-melting (with Δ*T*_1/2_ up to 9.8 °C; Additional file 1: Fig. S6) and UV-melting (with Δ*T*_1/2_ up to 5.9 °C; Additional file 1: Fig. S7). N-TASQ is thus G4-affinic but also highly G4-specific (being uniquely actively selective for G4s owing to their smart nature); the results seen in Fig. 1E (and Additional file 1: Figure S4), with a ratio of fluorescence F/F_0_ between 4 and 6 in all instances confirmed the G4 formation in these lncRNAs (even *LINC01589*, which shows that this possible mixture contains majorly G4s). These results align with those recently collected in a similar experimental setup (96-well format) obtained with both DNA and RNA G4s (SRC and VEGF, respectively), for which F/F_0_ up to 10 were obtained [75]. These results were confirmed using another well-established G4 turn-on probe thioflavin T (ThT) [76, 77]: as seen in Additional file 1: Fig. S8, the high F/F_0_ ratios obtained during these titrations provide a strong argument in support of the G4 fold of *UXT-AS1* (F/F_0_ = 162-fold), *MELTF-AS1* (161-fold), and *LINC01589* (96-fold). Next, we implemented Förster resonance energy transfer-melting competition (FRET-MC) assay [78], using the firmly established G4 F21T (a 21-nucleotide, human telomere-mimicking sequence d[^5′^G_3_(T_2_AG_3_)_3_^3′^] labeled with a fluorescein (F) on its 5′-end and a tetramethylrhodamine (T) on its 3′-end), stabilized by N-TASQ: the mid-transition temperature (*T*_1/2_) of N-TASQ-stabilized F21T is 67.9 °C; in the presence of a competitive G4, this *T*_1/2_ value should decrease, as a result of the distribution of N-TASQ on both labeled and unlabeled G4s. The strong decrease observed in the presence of *LINC01589*, *MELTF-AS1*, and *UXT-AS1* lncRNAs (Δ*T*_1/2_ = − 17.3, − 18.1, and − 18.2 °C, respectively) fully supports the formation of G4s in these lncRNAs (Fig. 1F). The thermal stability of these G4s was also confirmed by both CD-melting and UV-melting experiments, with *T*_1/2_ = 54.9, 60.0, and 72.4 °C (Additional file 1: Fig. S6) and 50.9, 61.3, and 70.2 °C (Additional file 1: Fig. S7) for the G4s formed in *LINC01589*, *MELTF-AS1*, and *UXT-AS1* lncRNAs, respectively, in line with their cognate 2G-, 3G-, and 4G-G4 nature. Of note, the CD-melting profile of *UXT-AS1* exhibited a biphasic pattern with a weak first transition, suggesting the presence of two distinct structural species in solution. Finally, the molecularity of these G4s was assessed by mass spectrometry (MS), using a combination of hydrophilic interaction liquid chromatography (HILIC) and electrospray ionization mass spectrometry (ESI–MS) analyses [79, 80] and peaks detected corresponded to the molecular weight of intramolecular G4s (14.5, 13.6 and 13.6 kDa for *LINC01589*, *MELTF-AS1*, and *UXT-AS1*, respectively) (Additional file 1: Fig. S9). Of note, the MS profile of *UXT-AS1* showed an additional peak, confirming the CD-melting results implying the presence of two distinct structural species. This set of results thus concurred in demonstrating the G4-folding of the PQSs found in the *LINC01589, MELTF-AS1*, and *UXT-AS1* lncRNAs.

### *LINC01589*, *MELTF-AS1*, and *UXT-AS1* lncRNAs fold into G4s in CRC cells

We next investigated the formation of *LINC01589*, *MELTF-AS1*, and *UXT-AS1* lncRNA G4s in CRC cells. We selected HT-29 as a model cell line for our investigations, as the expression levels of these three lncRNAs were found to be similar (Fig. 1C): this selection indeed removed a possible bias related to the initial abundance of lncRNA in our subsequent analyses, bearing in mind that it does not prejudge in any way that their G4s do actually fold in cells.

**Fig. 2:**
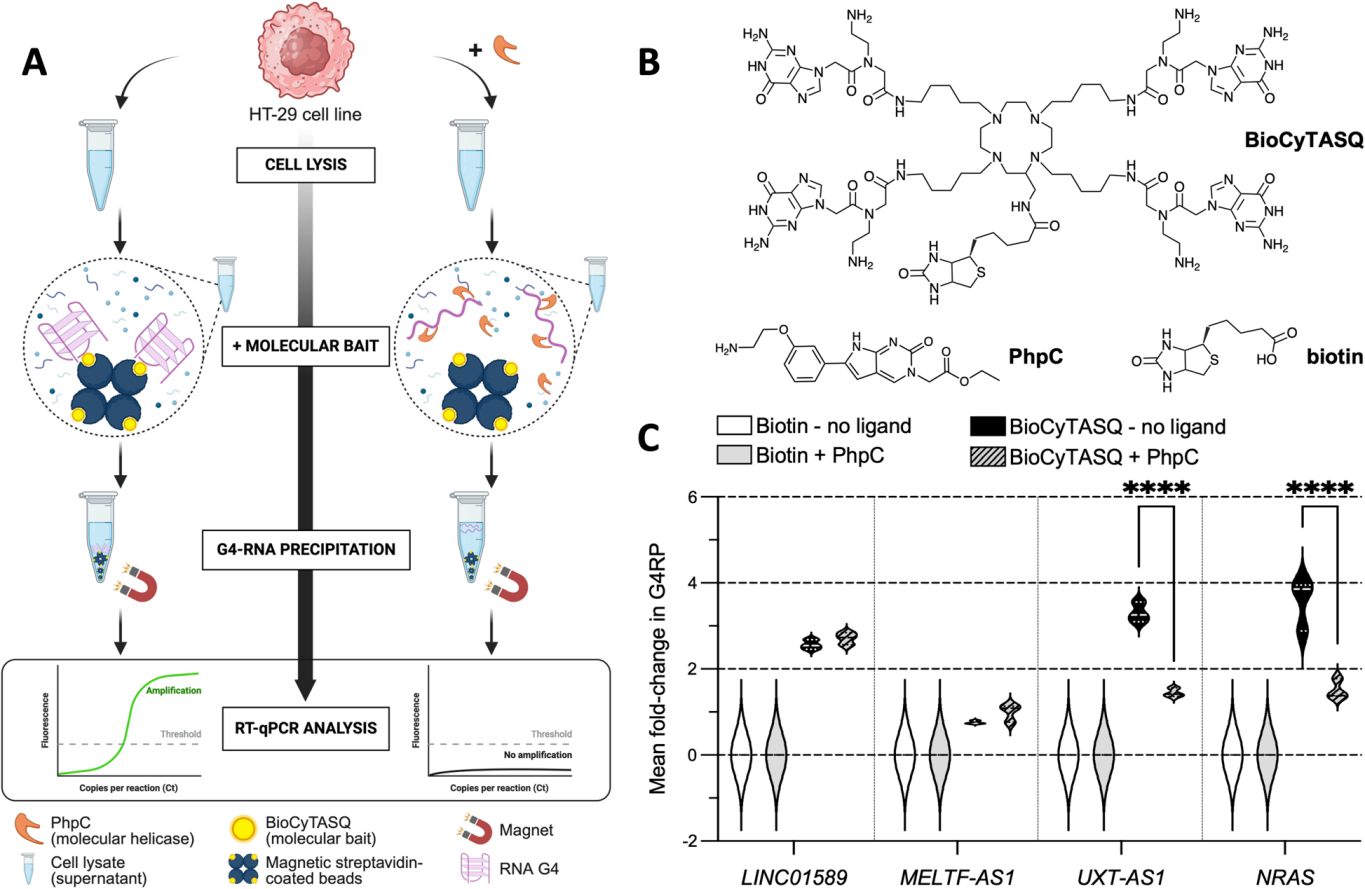
**A** Principle of the G4-RNA-specific precipitation (G4RP) protocol coupled with RT-qPCR (G4RP-RT-qPCR). Created with BioRender (Monchaud, D. (2025) https://BioRender.com/7if50um). **B** Chemical structure of the molecular helicase PhpC, the molecular bait BioCyTASQ and biotin, used as control. **C**. Results of the G4RP-RT-qPCR performed with HT-29 cells either untreated or treated with PhpC (90 μM). Mean ± SD fold-change in the level of RNAs [5 * {2^(Mean Ct input – Ct G4RP or biotin)^}]. The results are collected from triplicates (*n* = 3) across three independent studies (*n* = 3). Two-way ANOVA was applied for the statistical analyses, with *: *P* ≤ 0.05, **: *P* ≤ 0.01, ***: *P* ≤ 0.001, ****: *P* ≤ 0.0001; non-significant *P*-values are not represented

To this end, we implemented the G4RP protocol coupled with RT-qPCR analyses (G4RP-RT-qPCR, Fig. 2A, Additional file 3: Table S2) [54, 55]: the HT-29 cells were fixed with paraformaldehyde (to capture transiently folded G4s), lysed, and the resulting lysate was incubated with BioCyTASQ, which serves as a molecular bait for pulling G4s down. BioCyTASQ also belongs to the family of TASQs, its central template being a biotinylated macrocycle (Fig. 2B). As above, it interacts with DNA and RNA G4s with a ^app^*K*_D_ in between 0.5 and 1.0 mM (Additional file 1: Fig. S5) [57, 73, 81] and we further showed here that it interacts with *LINC01589*, *MELTF-AS1*, and *UXT-AS1* G4s in vitro by both CD-melting and UV-melting: as seen in Additional file 1: Figs. S6 and S7, a fair-to-good stabilization was obtained (Δ*T*_1/2_ up to 11.0 °C in CD, and to 12.4 °C in UV), confirming its good G4-stabilization. For the G4RP investigations, we used biotin as a negative molecular bait control, and *NRAS* mRNA as a positive RNA G4 control because (i) HT-29 cells are known to be *NRAS* wild-type [82], (ii) *NRAS* mRNA is known to be G4-rich [83], and (iii) the G4RP protocol was calibrated using *NRAS* G4 [53, 55, 57]. The RT-qPCR analyses of the BioCyTASQ-precipitated and purified RNAs revealed a 2.56-, 0.75-, and 3.30-fold change for the *LINC01589*, *MELTF-AS1*, and *UXT-AS1*, respectively, along with a 3.56-fold change for *NRAS* mRNA, as previously reported [55, 57], and no fold change when using biotin (Fig. 2C). These values, which are normalized *versus* the input (that is, untreated RNA samples), cannot be exploited to infer the natural abundance of the lncRNA in HT-29 cells *per se* but solely the modulation of their abundance upon cell treatment with G4-interacting compounds (*vide infra*). These results thus strongly support the existence of G4s in the *LINC01589* and *UXT-AS1* lncRNAs in CRC cells; the question of the existence of cellular *MELTF-AS1* G4 remains open at this stage.

### *LINC01589*, *MELTF-AS1*, and *UXT-AS1* G4s are targetable in CRC cells

With the existence of some of these lncRNA G4s established in cells, a strategy to fight against CRC would be to perturb their biological functions by targeting their G4s: however, contrarily to classical approaches using G4 stabilizers (ligands) [84, 85], we believe that ironing out RNA G4s using G4 destabilizers could be a more efficient approach [86, 87]. The reason is twofold: first, because of the serious adverse effects related to the use of G4 ligands, chiefly an indiscriminate genetic instability (DNA damage) [39–41, 88], which is good for treating cancer cells but must be avoided for surrounding cells and tissues. Second, because if these G4s present in dysregulated lncRNAs are detectable in CRC cells (*vide supra*), they likely play an active role in the oncogenic process (*e.g*., acting as binding hubs for RBPs): therefore, an original way to impair this pathological activity would be to unfold them and assess whether this treatment might disrupt the tumorigenic development. However, as discussed above [26], while the relevance of this G4-unfolding strategy was cleared using TMPyP4, its debatable G4-stabilizing *versus* G4-destabilizing properties make this validation questionable [86]. This is why, among the handful of G4-destabilizers reported to date [86, 87], we used PhpC as its properties have already been investigated in different human cell lines, including cancer (MCF7 [57], HeLa [89], and A549 cells [90]) and central nervous system (CNS) cells (patient-derived astrocytes [58] and neural progenitor cells [91]). We first checked by both CD-melting and UV-melting the interaction between PhpC and lncRNA G4s, having in mind that variable-temperature techniques are not fully suited to this purpose (being generally used to discard G4-stabilizers) [56]: as seen in Additional file 1: Figs. S6 and S7, a thermal destabilization was observed only for *UXT-AS1* in UV-melting (Δ*T*_1/2_ = − 1.0 °C). Next, we used a non-toxic concentration of PhpC (90 μM, its IC_50_ established by the SRB assay [92] being 242 μM after a 72-h treatment in HT-29, Additional file 1: Fig. S10) and assessed the resulting modulation of RNAs by G4RP-RT-qPCR analyses (Additional file 3: Table S2): no significant effects were observed with PhpC against *LINC01589* and *MELTF-AS1*, while PhpC triggered a significant 1.85- and 2.05-fold decrease against *UXT-AS1* and *NRAS*, respectively (Fig. 2C), the latter being in line with previously reported results. These results thus show that the cellular abundance of highly stable G4s (such as those found in *UXT-AS1* lncRNA and *NRAS* mRNA) can be modulated by ad hoc molecular effectors: while indirect effects cannot be ruled out, these results embrace a dynamic of G4-modulation by PhpC, which has found applications in both human cancer and CNS cells (*vide supra*). This emerging strategy of G4-destabilization, which remains to be consolidated, opens dazzling perspectives to mitigate G4-associated diseases. However, the fact that PhpC operates on some RNA G4s (*UXT-AS1* and *NRAS*) but not on other G4s (*LINC01589* and *MELTF-AS1*) indicate that caution must be exercised when interpreting these data, as PhpC might modulate their abundance upon interaction with yet-to-be-discovered structural features. This was confirmed during our exploration of G4-stabilization strategy using the well-established G4 ligand BRACO-19, selected here as it was used in the initial G4RP investigations [22, 53, 55, 57]. We first checked by both CD-melting and UV-melting the interaction between BRACO-19 and lncRNA G4s: as seen in Additional file 1: Figs. S6 and S7, a notable stabilization was obtained in all instance (Δ*T*_1/2_ up to 7.1 °C in CD and to 9.8 °C in UV), confirming its good G4-stabilization. Next, we used a non-toxic concentration of BRACO-19 (10 μM, its IC_50_ being 20.2 μM after a 72-h treatment in HT-29 cells, Fig. S10) and assessed the resulting modulation of RNAs by G4RP-RT-qPCR analyses (Fig. S11): as above, only the abundance of *UXT-AS1* and the control *NRAS* was significantly modulated, confirming that these two RNA G4s are more responsive to small molecule treatment than *LINC01589* and *MELTF-AS1*. However, while the results obtained with *NRAS* were in line with previously reported data (BRACO-19 triggers a 1.58-fold increase in *NRAS* abundance) [55, 57], the decrease observed with *UXT-AS1* (1.48-fold) indicated that both G4-interacting molecules, BRACO-19 and BioCyTASQ, might compete for binding to this particular—and unique—G4 structure (they display comparable affinity for DNA/RNA G4s (with ^app^*K*_D_ = 0.4–1.0 mM, determined by FQA, Additional file 1: Fig. S5) [57, 73], which is not the case with PhpC (^app^*K*_D_ > 100 mM)). However, combined with its ability to trigger genetic instability (notably at telomeres) [93], the use of BRACO-19 might thus be avoided, as its antiproliferative activity might originate in a series of non-predictable, certainly pleiotropic effects: this could yield real therapeutic dividends [94] (if a therapeutic window can be found) but makes the elucidation of its mechanism of action quite challenging.

## Conclusions

Advancements in high-throughput techniques for analysing human transcriptome have revealed the involvement of lncRNAs in key biological processes and their association with various pathophysiological conditions [95]. Despite the established connection between lncRNAs dysregulation and diseases, such as cancers, the role of their secondary structures in modulating disease progression remains relatively unexplored [7–10]. Although CRC ranks high in cancer-related mortalities, not much is known about the molecular basis of its carcinogenesis. This explains why, over the last ten years, numerous studies have focused on dysregulated lncRNAs in CRC [44–46]. Recently, a few G4-harboring lncRNAs have been linked with CRC modulation, highlighting the possible roles of G4s as hubs for various CRC-associated regulatory entities [47–49]. However, the lack of understanding regarding G4-formation in other dysregulated lncRNAs and the overall influence of their G4s on CRC progression led us to explore the G4-formation in additional CRC-dysregulated lncRNAs. Using in silico tools along with in vitro and cell-based assays, we demonstrate that the CRC-dysregulated *LINC01589*, *MELTF-AS1*, and *UXT-AS1* lncRNAs (i) are expressed in CRC, and other cancers as well, suggesting their probable role in diverse cancers; (ii) form stable G4s in vitro; (iii) harbor G4s that are detectable in CRC cells, notably for *LINC01589* and *UXT-AS1*; (iv) harbor G4s that can be efficiently targeted by the prototype molecule PhpC in CRC cells, notably for *UXT-AS1*. This latter point opens interesting therapeutic opportunities: indeed, a classical strategy would have been to target lncRNA G4s using G4 stabilizers (ligands). However, we discouraged this approach as we showed that G4 ligands can be responsible for serious adverse effects when crossing the blood–brain barrier and triggering DNA damage in central nervous system (CNS) cells for instance [96–99]. Instead, we have highlighted an innovative strategy hinging on the destabilization of G4s using PhpC, taking into account that if G4s are folded in a tumorous context, their unfolding might disturb the inner functions of cancer cells. We must stress that the results gathered in this study have demonstrated the existence of G4s in the selected lncRNAs, but the roles that these structures may play in lncRNA biology in general, and in the oncogenetic process in particular, remain to be deciphered. Should these structures serve as docking sites for key protein effectors (thus controlling subsequent effects), their disruption with ad hoc molecules would represent a novel and, above all, safe way to perturb tumor proliferation, as it does not rely on the use of genotoxic compounds, as is the case for the G4 ligands (PDS, TMPyP4, BRACO-19) used in aforementioned studies. We have demonstrated here that such a strategy is feasible using PhpC but have also emphasized that massive efforts must now be invested to uncover the way it acts both in vitro (e.g., what are the structural features that drive PhpC/G4 interactions? How could it distort the structure of a G4-prone sequence?) and in cells (e.g., what is the necessary dynamic for a G4 to be unfolded in a cellular context? Is a coordination with G4-binding proteins required for G4 destabilization?). It is critical to note that PhpC could interact with many cellular G4s, which is precisely at the origin of its genoprotective effects in CNS cells (for instance, it affects the expression of > 300 proteins in patient-derived astrocytes, 94% of which being upregulated) [58]. We do believe that ironing out cancer-specific RNA G4s might strengthen its sheltering effect; however, a limitation of this approach is that it requires longer timescales to be efficient, in a manner that is reminiscent of the cancer cell remortalization mechanism that underlies the therapeutic use of drugs targeting telomerase for instance (which requires > 20 days of treatment to be effective) [100]. We thus believe that triggering small defects in cancer cells, rather than extensive DNA damage, might be the surest way to put a damper on their fate selectively, owing to their flawed repertoire of correction and/or compensatory mechanisms. Studies are currently ongoing (*i.e.*, dose, schedule, treatment duration, etc.) to assess whether this new way of disrupting the homeostasis of cancer cells might pave a *hitherto* uncharted path of anticancer strategies, and we hope to report on these results at the earliest opportunity.

## Methods

### *In silico* identification of putative G-quadruplex-forming sequences (PQS) in CRC-dysregulated lncRNAs

The Lnc2Cancer 3.0 database was used to obtain the list of all the lncRNAs dysregulated in CRC [41]. Subsequently, the FASTA sequences of all these lncRNAs and their transcript variants were retrieved from the NCBI Nucleotide database (genomic context: Genome Reference assembly GRCh38.p14 (GCF_000001405.40-RS_2024_08, 2022), Aug 23, 2024) [60]. The QGRS mapper tool was used to identify PQS within the sequences of the selected lncRNAs and their transcript variants. The parameters used for QGRS mapper were max length: 45; min G-group: 2; loop size: 0 to 36 [61]. The G4Hunter tool was used with the following parameters: window size: 45; threshold: 0.9 [62, 63]. The highest-scoring PQS within the selected lncRNAs were chosen for in vitro investigations (Additional file 2: Table S1; Additional file 4: Table S3).

### RNA isolation and reverse transcription-quantitative polymerase chain reaction (RT-qPCR)

RNA isolation from the mentioned cell lines was performed using the conventional TRIzol method. Following the RNA purification and DNase treatment of the purified RNAs, reverse transcription was carried out using the SuperScript™ III Reverse Transcriptase, Random Hexamers and Oligo(dT)_20_ Primer, following the manufacturer’s protocol. The resulting cDNAs were used to perform the quantitative Polymerase Chain Reaction (qPCR) with the iTaq Universal SYBR Green Supermix, as per the manufacturer’s protocol. The qPCR was carried out in the presence of primer pairs specific for the selected lncRNAs and housekeeping genes (GAPDH and β-Actin) (Additional file 3: Table S2) using the Mx3005P qPCR System. The data were recorded in triplicates across two independent studies to determine lncRNA expression levels in CRC (HT-29, HCT-116), breast cancer (HCC-1954, MDA-MB-231, MCF7), prostate cancer (PC3) and cervical cancer (HeLa) cell lines. The mean relative lncRNA expression (2^−ΔCt^) with respect to GAPDH was plotted with standard error of mean against different cancer cell lines using GraphPad Software. For assessing lncRNA expression levels in different CRC cell lines (HT-29, HCT-116, HCT-15, COLO205, COLO320DM and SW620), the data were recorded in triplicate from three independent studies. The mean relative lncRNA expression (2^−ΔCt^) with respect to β-Actin were plotted with standard deviation against different CRC cell lines using GraphPad Software. RT-qPCR from different cancer and CRC cell lines was performed in two separate laboratories, employing the housekeeping genes GAPDH and β-Actin, available in the respective laboratories.

### Circular dichroism (CD) and UV spectroscopy

For CD and UV experiments, spectra were recorded on a JASCO J-815 spectropolarimeter in a 10-mm path-length quartz semi-micro cuvette (Starna). Experiments were recorded at the wavelengths range from 210 to 350 nm at 25 and then 95 °C (sensitivity: standard, DIT: 1 s, bandwidth: 0.5 nm, data pitch: 1.0 nm, scanning speed: 100 nm/min, accumulations: 2) and performed with folded RNAs (1 µM) in 10 mM lithium cacodylate buffer (pH 7.2) plus 10 mM KCl and 90 mM LiCl. The data were zeroed at 320 nm. The CD data were smoothened (with the Savitzky-Golay method and 20 points of window), using OriginPro, Version 2018 (OriginLab Corporation).

### CD-melting and UV-melting

For CD and UV-melting experiments, spectra were recorded on a JASCO J-815 spectropolarimeter in a 10-mm path-length quartz semi-micro cuvette (Starna). Experiments were recorded at several wavelengths (see below) (starting: 25 °C, sampling: 2.0 °C, target: 95 °C, ramp rate: 2.0 °C/min, wait: 0 s, sensitivity: standard, DIT: 1 s, bandwidth: 0.5 nm) and performed with folded RNAs (1 µM) in the presence of G4-interacting compounds (PhpC, N-TASQ, BioCyTASQ and BRACO-19, 5 µM) in 10 mM lithium cacodylate buffer (pH 7.2) plus 10 mM KCl and 90 mM LiCl. The CD signal was monitored at 268 nm for *LINC01589* and 263 nm for *MELTF-AS1* and *UXT-AS1*; the UV signal was monitored at 258, 257, and 256 nm for *LINC01589*, *MELTF-AS1*, and *UXT-AS1*, respectively). The data were smoothened (with the Savitzky-Golay method and 20 points of window) and normalized (0–100) (for CD-melting) or only normalized (0–100) (for UV-melting), using OriginPro, Version 2018 (OriginLab Corporation). The mid-transition temperatures (Δ*T*_1/2_) were determined manually in order to characterize the ligands’ ability to interact with G4s via a modification of the Δ*T*_1/2_.

### Thermal difference spectra

TDS signatures were calculated by subtracting the UV spectra collected at 25 °C from those collected at 95 °C and then zeroed at 320 nm. Spectra were recorded on a JASCO V630Bio spectrophotometer in a 10 mm path-length quartz semi-micro cuvette (Starna). Spectra of 1 µM of RNA sample in 100 µL (final volume) of 10 mM lithium cacodylate buffer (pH 7.2) plus 10 mM KCl and 90 mM LiCl. Final data were treated with OriginPro, Version 2018 (OriginLab Corporation).

### N-TASQ fluorescence enhancement assay

RNAs (2 µM) folded in the supplementation of 10 mM KCl were titrated with N-TASQ (1 µM), reaching a final concentration of 5 µM. After each titration, the fluorescence emission spectrum of N-TASQ was recorded from 335 to 600 nm, along with endpoint fluorescence emission at 393 nm, with excitation at 280 nm using a CLARIOstar® Plus multimode plate reader (BMG LABTECH SARL, France) [68]. The data were recorded in duplicate as part of an independent study (due to RNA scarcity). The mean fluorescence intensities (arbitrary units) of emission spectra were plotted against the wavelength after smoothening the data with the Savitzky-Golay method and 20 points of window using Origin (Pro). The mean N-TASQ fluorescence fold-enhancements (endpoint fluorescence in the presence of RNA/absence of RNA: F/F_0_) were plotted with standard deviation against respective RNAs using GraphPad Software. Two-way ANOVA were employed for the statistical analyses.

### Thioflavin T (ThT) fluorescence enhancement assay

Folded RNAs (2 µM) were mixed with ThT (2 µM) and the ThT fluorescence emission spectra from 470 to 650 nm were recorded with excitation at 445 nm using a BioTek Cytation Hybrid Multimode Reader (Agilent Technologies International Pvt. Ltd., Manesar, India). The data were recorded in triplicates across two independent studies. The mean fluorescence intensities (arbitrary units) of emission spectra were plotted against the wavelength after smoothening the data with the Savitzky-Golay method and 20 points of window using Origin (Pro), Version 2017 (OriginLab Corporation, USA).

### Forster resonance energy transfer-melting competition (FRET-MC) assay

RNAs (3 µM) folded in the supplementation of 10 mM KCl were mixed with the folded F21T (0.2 µM) and N-TASQ (1 µM) in folding buffer: 10 mM Tris–HCl (pH 7.5) and 0.1 mM EDTA (pH 8.0), supplemented with 10 mM KCl. The thermal denaturation of this mixture was carried out from 25 °C to 90 °C, with the intensity of the FAM emission simultaneously recorded at intervals of 1 °C when excited at 488 nm, using a Mx3005P qPCR System (Agilent Technologies, France) [78]. The data were recorded in duplicates across two independent studies. The means of normalized FAM emission at maxima from N-TASQ-stabilized F21T in the presence or absence of RNAs were plotted with standard error of mean against the temperature for respective RNAs using GraphPad Software. The plot was fitted using the Boltzmann sigmoidal model of non-linear regression to determine the melting temperatures (*T*_1/2_) of the respective RNAs.

### G4-RNA-specific precipitation coupled with reverse transcription-quantitative polymerase chain reaction (G4RP-RT-qPCR)

The HT-29 cells were seeded at 8 × 10^6^ density in a T-175 tissue culture flask and cultured at 37 °C in the presence of 5% CO_2_ for 72 h. The cells were treated with PhpC (90 μM) or left untreated after 24 h of seeding. After 48 h of PhpC treatment or no treatment, the G4RP-RT-qPCR procedure was carried out as mentioned in the recent reports [22, 53–55, 58]. Subsequently, the cells were trypsinized, fixed with 1% paraformaldehyde, and lysed using a 27G (0.4 mm) needle and syringe. The cell lysate was centrifuged to collect the supernatant of the cell lysate. The resulting cell lysate (supernatant) was then incubated with biotinylated TASQ (BioCyTASQ) [81] (100 µM) or biotin (negative control) (100 µM) in the presence of Streptavidin MagneSphere® Paramagnetic Particles (150 µg) at 4 °C for 120 min. After washing the BioCyTASQ or Biotin-conjugated streptavidin beads, reverse crosslinking was carried out for both the input lysate and the bound streptavidin beads at 70 °C for 120 min. Following this, the input lysate and the bound streptavidin beads were snap-frozen in TRIzol™ Reagent. RNA isolation from the input lysate and the bound streptavidin beads was performed using the conventional TRIzol method. Following the in-column RNA purification and DNase treatment of the purified RNAs, RT-qPCR was carried out in the presence of primer pairs specific for the selected RNAs (Additional file 3: Table S2), as described earlier. The data were recorded in triplicates across three independent studies. The mean fold-change in the level of respective RNAs in BioCyTASQ (G4RP) precipitated or biotin samples relative to the input (5%) cell lysate in the presence or absence of PhpC treatment [5 * {2^(Mean Ct input – Ct G4RP or biotin)^}] was plotted with standard error of the mean using GraphPad Software. Two-way ANOVA was applied for the statistical analyses.

### Statistical analyses

Data were analyzed with Excel (Microsoft Corp.) and GraphPad Prism version 9.5.1 for Mac OS (GraphPad software) or OriginPro, version 2018 (OriginLab Corporation) and subjected to two-way ANOVA variance tests, with the statistical significance (*P*-values) ≤ 0.05, *P* ≤ 0.01, *P* ≤ 0.001, and *P* ≤ 0.0001 denoted with one asterisk (*), two asterisks (**), three asterisks (***), and four asterisks (****), respectively. Non-significant *P*-values are not represented.

## Supplementary Information

Additional file 1: details about 1-the selection of lncRNA using QGRS mapper [61] and G4Hunter [63], 2- the preparation of the oligonucleotides notably primers using primer-BLAST [101], 3- the reagents used in this study, 4- the cell lines and related culture protocols, 5- the ESI–MS protocol [102, 103] and 6- the SRB method [92]. Figure S1: Identification of Putative Quadruplex-forming Sequences (PQS) in long non-coding RNAs (lncRNAs) dysregulated in Colorectal cancer (CRC). Figure S2: Expression of LINC01589, MELTF-AS1, and UXT-AS1 lncRNAs in different cancer cell lines. Figure S3: CD and UV spectra, along with thermal difference signature (TDS) [65] calculated for LINC01589, MELTF-AS1, and UXT-AS1 lncRNAs. Figure S4: in vitro formation of stable G4s in LINC01589, MELTF-AS1, and UXT-AS1 lncRNAs assessed via N-TASQ fluorescence enhancement assay. Figure S5: fluorescence quenching assay (FQA) [74] to determine the affinity of G4-interacting molecules for either DNA G4 (Cy5-Myc) or RNA G4 (Cy5-NRAS). Figure S6: CD-melting experiments performed lncRNA G4s in the presence of ligands. Figure S7: UV-melting experiments performed with lncRNA G4s in the presence of ligands. Figure S8: Fluorescence titrations were performed with RNA G4s and ThT. Figure S9. ESI–MS analysis of folded synthetic RNA G4s. Figure S10: Cytotoxicity of PhpC and BRACO-19 in HT-29 cells using the SRB test. Figure S11. G4RP-RT-qPCR results [57, 73] obtained with BRACO19 and PhpC on LINC01589, MELTF-AS1, and UXT-AS1 lncRNA G4s in HT-29 cells.

Additional file 2: Table S1: Colorectal cancer-LncRNA G4s data.

Additional file 3: Table S2: RT-qPCR primers.

Additional file 4: Table S3: DNA and RNA oligonucleotides.

## Data Availability

All data generated or analysed during this study are included in this published article and its supplementary information files.

## Abbreviations

CD: Circular dichroism
CNS: Central nervous system
CRC: Colorectal cancer
ESI-MS: Electrospray ionization mass spectrometry
FRET: Förster resonance energy transfer
G4: G-quadruplex
G4RP: G4-RNA-specific precipitation
HILIC: Hydrophilic interaction liquid chromatography
lncRNA: Long non-coding RNA
NCBI: National Center for Biotechnology Information
NONO: Non-POU domain-containing octamer binding protein
PDS: Pyridostatin
PhpC: Phenylpyrrolocytosine
PQS: Putative G-quadruplex-forming sequence
QGRS: Quadruplex forming G-rich sequences
RBP: RNA binding protein
RT-qPCR: Reverse transcriptase quantitative polymerase chain reaction
SD: Standard deviation
TASQ: Template-assembled synthetic G-quartet
TDS: Thermal difference spectra

## References

[1] Iyer MK, Niknafs YS, Malik R, Singhal U, Sahu A, Hosono Y, et al. The landscape of long noncoding RNAs in the human transcriptome. Nat Genet. 2015;47(3):199–208. 25599403 10.1038/ng.3192 PMC4417758

[2] Mattick JS, Amaral PP, Carninci P, Carpenter S, Chang HY, Chen LL, et al. Long non-coding RNAs: definitions, functions, challenges and recommendations. Nat Rev Mol Cell Biol. 2023;24(6):430–47. 36596869 10.1038/s41580-022-00566-8 PMC10213152

[3] Fatica A, Bozzoni I. Long non-coding RNAs: New players in cell differentiation and development. Nat Rev Genet. 2014;15(1):7–21. 24296535 10.1038/nrg3606

[4] Zhang X, Wang W, Zhu W, Dong J, Cheng Y, Yin Z, et al. Mechanisms and functions of long non-coding RNAs at multiple regulatory levels. Int J Mol Sci. 2019;20(22):5573. 31717266 10.3390/ijms20225573 PMC6888083

[5] Statello L, Guo CJ, Chen LL, Huarte M. Gene regulation by long non-coding RNAs and its biological functions. Nat Rev Mol Cell Biol. 2020;22(2):96–118. 33353982 10.1038/s41580-020-00315-9 PMC7754182

[6] Chillón I, Marcia M. The molecular structure of long non-coding RNAs: emerging patterns and functional implications. Crit Rev Biochem Mol Biol. 2020;55(6):662–90. 33043695 10.1080/10409238.2020.1828259

[7] Sana J, Faltejskova P, Svoboda M, Slaby O. Novel classes of non-coding RNAs and cancer. J Transl Med. 2012;10(1):103. 22613733 10.1186/1479-5876-10-103 PMC3434024

[8] Arun G, Diermeier SD, Spector DL. Therapeutic Targeting of Long Non-Coding RNAs in Cancer. Trends Mol Med. 2018;24(3):257. 29449148 10.1016/j.molmed.2018.01.001 PMC5840027

[9] Ahmad M, Weiswald LB, Poulain L, Denoyelle C, Meryet-Figuiere M. Involvement of lncRNAs in cancer cells migration, invasion and metastasis: cytoskeleton and ECM crosstalk. J Exp Clin Cancer Res. 2023;42(1):173. 37464436 10.1186/s13046-023-02741-x PMC10353155

[10] Mo Y, Adu-Amankwaah J, Qin W, Gao T, Hou X, Fan M, et al. Unlocking the predictive potential of long non-coding RNAs: a machine learning approach for precise cancer patient prognosis. Ann Med. 2023;55(2):2279748. 37983519 10.1080/07853890.2023.2279748 PMC11571739

[11] Kharel P, Ivanov P. RNA G-quadruplexes and stress: emerging mechanisms and functions. Trends Cell Biol. 2024;34(9):771–84. 38341346 10.1016/j.tcb.2024.01.005 PMC12069074

[12] Sahayasheela VJ, Sugiyama H. RNA G-quadruplex in functional regulation of noncoding RNA: Challenges and emerging opportunities. Cell Chem Biol. 2023;31(1):53–70. 37909035 10.1016/j.chembiol.2023.08.010

[13] Li F, Zhou J. G-quadruplexes from non-coding RNAs. J Mol Med. 2023;101(6):621–35. 37069370 10.1007/s00109-023-02314-7

[14] Lyu K, Chow EYC, Mou X, Chan TF, Kwok CK. RNA G-quadruplexes (rG4s): genomics and biological functions. Nucleic Acids Res. 2021;49(10):5426–50. 33772593 10.1093/nar/gkab187 PMC8191793

[15] Tassinari M, Richter SN, Gandellini P. Biological relevance and therapeutic potential of G-quadruplex structures in the human noncoding transcriptome. Nucleic Acids Res. 2021;49(7):3617–33. 33721024 10.1093/nar/gkab127 PMC8053107

[16] Dumas L, Herviou P, Dassi E, Cammas A, Millevoi S. G-Quadruplexes in RNA Biology: Recent Advances and Future Directions. Trends Biochem Sci. 2021;46(4):270–83. 33303320 10.1016/j.tibs.2020.11.001

[17] Kharel P, Becker G, Tsvetkov V, Ivanov P. Properties and biological impact of RNA G-quadruplexes: From order to turmoil and back. Nucleic Acids Res. 2020;48(22):12534–55. 33264409 10.1093/nar/gkaa1126 PMC7736831

[18] Fay MM, Lyons SM, Ivanov P. RNA G-Quadruplexes in Biology: Principles and Molecular Mechanisms. J Mol Biol. 2017;429(14):2127–47. 28554731 10.1016/j.jmb.2017.05.017 PMC5603239

[19] Jayaraj GG, Pandey S, Scaria V, Maiti S. Potential G-quadruplexes in the human long non-coding transcriptome. RNA Biol. 2012;9(1):81–6. 22258148 10.4161/rna.9.1.18047

[20] Yang SY, Lejault P, Chevrier S, Boidot R, Robertson AG, Wong JM, et al. Transcriptome-wide identification of transient RNA G-quadruplexes in human cells. Nat Commun. 2018;9(1):4730. 30413703 10.1038/s41467-018-07224-8 PMC6226477

[21] Kwok CK, Marsico G, Sahakyan AB, Chambers VS, Balasubramanian S. rG4-seq reveals widespread formation of G-quadruplex structures in the human transcriptome. Nat Meth. 2016;13(10):841–4. 10.1038/nmeth.3965 27571552

[22] Yang SY, Monchaud D, Wong JMY. Global mapping of RNA G-quadruplexes (G4-RNAs) using G4RP-seq. Nat Protoc. 2022;17(3):870–89. 35140410 10.1038/s41596-021-00671-6

[23] Yu B, Shan G. Functions of long noncoding RNAs in the nucleus. Nucleus. 2016;7(2):155–66. 27105038 10.1080/19491034.2016.1179408 PMC4916869

[24] Wheelhouse RT, Sun D, Han H, Han FX, Hurley LH. Cationic Porphyrins as Telomerase Inhibitors: the Interaction of Tetra-(N-methyl-4-pyridyl)porphine with Quadruplex DNA. J Am Chem Soc. 1998;120(13):3261–2.

[25] Rodriguez R, Mueller S, Yeoman JA, Trentesaux C, Riou J-F, Balasubramanian S. A Novel Small Molecule That Alters Shelterin Integrity and Triggers a DNA-Damage Response at Telomeres. J Am Chem Soc. 2008;130(47):15758. 18975896 10.1021/ja805615w PMC2746963

[26] Simko EA, Liu H, Zhang T, Velasquez A, Teli S, Haeusler AR, et al. G-quadruplexes offer a conserved structural motif for NONO recruitment to NEAT1 architectural lncRNA. Nucleic Acids Res. 2020;48(13):7421–38. 32496517 10.1093/nar/gkaa475 PMC7367201

[27] Mou X, Liew SW, Kwok CK. Identification and targeting of G-quadruplex structures in MALAT1 long non-coding RNA. Nucleic Acids Res. 2022;50(1):397–410. 34904666 10.1093/nar/gkab1208 PMC8754639

[28] Ronchetti D, Traini V, Silvestris I, Fabbiano G, Passamonti F, Bolli N, et al. The pleiotropic nature of NONO, a master regulator of essential biological pathways in cancers. Cancer Gene Ther. 2024;31(7):984–94. 38493226 10.1038/s41417-024-00763-x PMC11257950

[29] Weisman-Shomer P, Cohen E, Hershco I, Khateb S, Wolfovitz-Barchad O, Hurley LH, et al. The cationic porphyrin TMPyP4 destabilizes the tetraplex form of the fragile X syndrome expanded sequence d (CGG) n. Nucleic Acids Res. 2003;31(14):3963–70. 12853612 10.1093/nar/gkg453 PMC165968

[30] Ofer N, Weisman-Shomer P, Shklover J, Fry M. The quadruplex r (CGG) n destabilizing cationic porphyrin TMPyP4 cooperates with hnRNPs to increase the translation efficiency of fragile X premutation mRNA. Nucleic Acids Res. 2009;37(8):2712–22. 19273535 10.1093/nar/gkp130 PMC2677883

[31] Haldar S, Zhang Y, Xia Y, Islam B, Liu S, Gervasio FL, et al. Mechanistic Insights into the Ligand-Induced Unfolding of an RNA G-Quadruplex. J Am Chem Soc. 2022;144(2):935–50. 34989224 10.1021/jacs.1c11248

[32] Zamiri B, Reddy K, Macgregor RB, Pearson CE. TMPyP4 porphyrin distorts RNA G-quadruplex structures of the disease-associated r (GGGGCC) n repeat of the C9orf72 gene and blocks interaction of RNA-binding proteins. J Biol Chem. 2014;289(8):4653–9. 24371143 10.1074/jbc.C113.502336 PMC3931028

[33] Siddiqui-Jain A, Grand CL, Bearss DJ, Hurley LH. Direct evidence for a G-quadruplex in a promoter region and its targeting with a small molecule to repress c-MYC transcription. Proc Natl Acad Sci U S A. 2002;99(18):11593–8. 12195017 10.1073/pnas.182256799 PMC129314

[34] Monchaud D, Granzhan A, Saettel N, Guédin A, Mergny J-L, Teulade-Fichou M-P. “One Ring to Bind Them All”—Part I: The Efficiency of the Macrocyclic Scaffold for G-Quadruplex DNA Recognition. J Nucleic Acids. 2010;2010:525862. 20725629 10.4061/2010/525862 PMC2915875

[35] Kwok CK, Sahakyan AB, Balasubramanian S. Structural Analysis using SHALiPE to Reveal RNA G-Quadruplex Formation in Human Precursor MicroRNA. Angew Chem. 2016;128(31):9104–7. 10.1002/anie.201603562 PMC6680278 27355429

[36] Santos T, Miranda A, Imbert L, Monchaud D, Salgado GF, Cabrita EJ, et al. Targeting a G-quadruplex from let-7e pre-miRNA with small molecules and nucleolin. J Pharm Biomed Anal. 2022;215: 114757. 35462282 10.1016/j.jpba.2022.114757

[37] Lejault P, Prudent L, Terrier M-P, Perreault J-P. Small molecule chaperones facilitate the folding of RNA G-quadruplexes. Biochimie. 2023;214:83–90. 37666291 10.1016/j.biochi.2023.08.016

[38] Rodriguez R, Miller KM, Forment JV, Bradshaw CR, Nikan M, Britton S, et al. Small-molecule-induced DNA damage identifies alternative DNA structures in human genes. Nat Chem Biol. 2012;8(3):301–10. 22306580 10.1038/nchembio.780 PMC3433707

[39] Olivieri M, Cho T, Álvarez-Quilón A, Li K, Schellenberg MJ, Zimmermann M, et al. A Genetic Map of the Response to DNA Damage in Human Cells. Cell. 2020;182(2):481-96.e21. 32649862 10.1016/j.cell.2020.05.040 PMC7384976

[40] Bossaert M, Pipier A, Riou J-F, Noirot C, Nguyên L-T, Serre R-F, et al. Transcription-associated topoisomerase 2α (TOP2A) activity is a major effector of cytotoxicity induced by G-quadruplex ligands. eLife. 2021;10:e65184. 34180392 10.7554/eLife.65184 PMC8279764

[41] Groelly FJ, Porru M, Zimmer J, Benainous H, De Visser Y, Kosova AA, et al. Anti-tumoural activity of the G-quadruplex ligand pyridostatin against BRCA1/2-deficient tumours. EMBO Mol Med. 2022;14(3):e14501. 35107878 10.15252/emmm.202114501 PMC8899905

[42] Ren Z, Tao Z. Molecular Basis of Colorectal Cancer: Tumor Biology. Surgical Treatment of Colorectal Cancer: Asian Perspectives on Optimization and Standardization. 2018:23–34.

[43] Nguyen LH, Goel A, Chung DC. Pathways of Colorectal Carcinogenesis. Gastroenterology. 2020;158(2):291–302. 31622622 10.1053/j.gastro.2019.08.059 PMC6981255

[44] Siddiqui H, Al-Ghafari A, Choudhry H, Al DH. Roles of long non-coding RNAs in colorectal cancer tumorigenesis: A review. Mol Clin Oncol. 2019;11(2):167–72. 31281651 10.3892/mco.2019.1872 PMC6589935

[45] Chen S, Shen X. Long noncoding RNAs: functions and mechanisms in colon cancer. Mol Cancer. 2020;19(1):167. 33246471 10.1186/s12943-020-01287-2 PMC7697375

[46] Irfan M, Javed Z, Khan K, Khan N, Docea AO, Calina D, et al. Apoptosis evasion via long non-coding RNAs in colorectal cancer. Cancer Cell Int. 2022;22(1):280. 36076273 10.1186/s12935-022-02695-8 PMC9461221

[47] Matsumura K, Kawasaki Y, Miyamoto M, Kamoshida Y, Nakamura J, Negishi L, et al. The novel G-quadruplex-containing long non-coding RNA GSEC antagonizes DHX36 and modulates colon cancer cell migration. Oncogene. 2016;36(9):1191–9. 27797375 10.1038/onc.2016.282

[48] Yari H, Jin L, Teng L, Wang Y, Wu Y, Liu GZ, et al. LncRNA REG1CP promotes tumorigenesis through an enhancer complex to recruit FANCJ helicase for REG3A transcription. Nat Commun. 2019;10(1):5334. 31767869 10.1038/s41467-019-13313-z PMC6877513

[49] Wu R, Li L, Bai Y, Yu B, Xie C, Wu H, et al. The long noncoding RNA LUCAT1 promotes colorectal cancer cell proliferation by antagonizing Nucleolin to regulate MYC expression. Cell Death Dis. 2020;11(10):908. 33097685 10.1038/s41419-020-03095-4 PMC7584667

[50] Guo Z, Zhou C, Zhong X, Shi J, Wu Z, Tang K, et al. The long noncoding RNA CTA-941F9.9 is frequently downregulated and may serve as a biomarker for carcinogenesis in colorectal cancer. J Clin Lab Anal. 2019;33(9):e22986. 31343781 10.1002/jcla.22986 PMC6868415

[51] Li C, Tan F, Pei Q, Zhou Z, Zhou Y, Zhang L, et al. Non-coding RNA MFI2-AS1 promotes colorectal cancer cell proliferation, migration and invasion through miR-574-5p/MYCBP axis. Cell Prolif. 2019;52(4): e12632. 31094023 10.1111/cpr.12632 PMC6668983

[52] Yin J, Luo W, Zeng X, Zeng L, Li Z, Deng X, et al. UXT-AS1-induced alternative splicing of UXT is associated with tumor progression in colorectal cancer. Am J Cancer Res. 2017;7(3):462. 28401004 PMC5385636

[53] Yang SY, Lejault P, Chevrier S, Boidot R, Robertson AG, Wong JMY, et al. Transcriptome-wide identification of transient RNA G-quadruplexes in human cells. Nat Commun. 2018;9(1):4730. 30413703 10.1038/s41467-018-07224-8 PMC6226477

[54] Renard I, Grandmougin M, Roux A, Yang SY, Lejault P, Pirrotta M, et al. Small-molecule affinity capture of DNA/RNA quadruplexes and their identification in vitro and in vivo through the G4RP protocol. Nucleic Acids Res. 2019;47(11):5502–10. 30949698 10.1093/nar/gkz215 PMC6582334

[55] Mitteaux J, Monchaud D. Protocol for cellular RNA G-quadruplex profiling using G4RP.v2. STAR Protoc. 2024;5(4):103480. 39661503 10.1016/j.xpro.2024.103480 PMC11697541

[56] Mitteaux J, Lejault P, Wojciechowski F, Joubert A, Boudon J, Desbois N, et al. Identifying G-Quadruplex-DNA-Disrupting Small Molecules. J Am Chem Soc. 2021;143(32):12567–77. 34346684 10.1021/jacs.1c04426

[57] Mitteaux J, Raevens S, Wang Z, Pirrotta M, Valverde IE, Hudson RHE, et al. PhpC modulates G-quadruplex-RNA landscapes in human cells. Chem Commun. 2024;60(4):424–7. 10.1039/d3cc05155b 38086624

[58] Vijay Kumar MJ, Mitteaux J, Wang Z, Wheeler E, Tandon N, Jung SY, et al. Small molecule-based regulation of gene expression in human astrocytes switching on and off the G-quadruplex control systems. J Biol Chem. 2024;0(0):108040. 10.1016/j.jbc.2024.108040 PMC11750478 39615684

[59] Gao Y, Shang S, Guo S, Li X, Zhou H, Liu H, et al. Lnc2Cancer 3.0: an updated resource for experimentally supported lncRNA/circRNA cancer associations and web tools based on RNA-seq and scRNA-seq data. Nucleic Acids Res. 2021;49(D1):D1251-D8. 10.1093/nar/gkaa1006 PMC7779028 33219685

[60] Sayers EW, Bolton EE, Brister JR, Canese K, Chan J, Comeau DC, et al. Database resources of the national center for biotechnology information. Nucleic Acids Res. 2022;50(D1):D20-D. 10.1093/nar/gkab1112 PMC8728269 34850941

[61] Kikin O, D’Antonio L, Bagga PS. QGRS Mapper: a web-based server for predicting G-quadruplexes in nucleotide sequences. Nucleic Acids Res. 2006;34:W676–82. 16845096 10.1093/nar/gkl253 PMC1538864

[62] Brázda V, Kolomazník J, Lýsek J, Bartas M, Fojta M, Šťastný J, et al. G4Hunter web application: a web server for G-quadruplex prediction. Bioinformatics. 2019;35(18):3493–5. 30721922 10.1093/bioinformatics/btz087 PMC6748775

[63] Bedrat A, Lacroix L, Mergny J-L. Re-evaluation of G-quadruplex propensity with G4Hunter. Nucleic Acids Res. 2016;44(4):1746–59. 26792894 10.1093/nar/gkw006 PMC4770238

[64] Vorlickova M, Kejnovska I, Sagi J, Renciuk D, Bednarova K, Motlova J, et al. Circular dichroism and guanine quadruplexes. Methods. 2012;57(1):64–75. 22450044 10.1016/j.ymeth.2012.03.011

[65] Mergny JL, Li J, Lacroix L, Amrane S, Chaires JB. Thermal difference spectra: a specific signature for nucleic acid structures. Nucleic Acids Res. 2005;33(16). 10.1093/nar/gni134 PMC1201377 16157860

[66] Stefan L, Monchaud D. Applications of guanine quartets in nanotechnology and chemical biology. Nat Rev Chem. 2019;3:650–68.

[67] Monchaud D. Template-Assembled Synthetic G-Quartets (TASQs): multiTASQing Molecular Tools for Investigating DNA and RNA G-Quadruplex Biology. Acc Chem Res. 2023;56:350–62. 36662540 10.1021/acs.accounts.2c00757

[68] Laguerre A, Hukezalie K, Winckler P, Katranji F, Chanteloup G, Pirrotta M, et al. Visualization of RNA-quadruplexes in live cells. J Am Chem Soc. 2015;137(26):8521–5. 26056849 10.1021/jacs.5b03413

[69] Yang SY, Amor S, Laguerre A, Wong JM, Monchaud D. Real-time and quantitative fluorescent live-cell imaging with quadruplex-specific red-edge probe (G4-REP). Biochim Biophys Acta. 2017;1861(5):1312–20. 10.1016/j.bbagen.2016.11.046 27956241

[70] Yang SY, Chang EYC, Lim J, Kwan HH, Monchaud D, Yip S, et al. G-quadruplexes mark alternative lengthening of telomeres. NAR Cancer. 2021;3(3):zcab031. 10.1093/narcan/zcab031 PMC8294677 34316718

[71] Laguerre A, Wong JMY, Monchaud D. Direct visualization of both DNA and RNA quadruplexes in human cells via an uncommon spectroscopic mechanism. Sci Rep. 2016;6:32141. 27535322 10.1038/srep32141 PMC4989495

[72] Zhou J, Roembke BT, Paragi G, Laguerre A, Sintim HO, Fonseca Guerra C, et al. Computational understanding and experimental characterization of twice-as-smart quadruplex ligands as chemical sensors of bacterial nucleotide second messengers. Sci Rep. 2016;6:33888. 27667717 10.1038/srep33888 PMC5036188

[73] Rota Sperti F, Mitteaux J, Zell J, Pipier A, Valverde IE, Monchaud D. The multivalent G-quadruplex (G4)-ligands MultiTASQs allow for versatile click chemistry-based investigations. RSC Chem Biol. 2023;4:456–65. 37415864 10.1039/d3cb00009e PMC10320843

[74] Le DD, Di Antonio M, Chan LKM, Balasubramanian S. G-quadruplex ligands exhibit differential G-tetrad selectivity. Chem Commun. 2015;51(38):8048–50. 10.1039/c5cc02252e 25864836

[75] Ledvinka J, Rota Sperti F, Paragi G, Pirrotta M, Chéron N, Valverde IE, et al. Fluorescence Detection of DNA/RNA G-Quadruplexes (G4s) by Twice-as-Smart Ligands. ChemMedChem. 2025;20(7):e202400829. 39714851 10.1002/cmdc.202400829 PMC11961149

[76] Mohanty J, Barooah N, Dhamodharan V, Harikrishna S, Pradeepkumar PI, Bhasikuttan AC. Thioflavin T as an Efficient Inducer and Selective Fluorescent Sensor for the Human Telomeric G-Quadruplex DNA. J Am Chem Soc. 2013;135(1):367–76. 23215453 10.1021/ja309588h

[77] Renaud de la Faverie A, Guedin A, Bedrat A, Yatsunyk LA, Mergny J-L. Thioflavin T as a fluorescence light-up probe for G4 formation. Nucleic Acids Res. 2014;42(8):e65-e. 10.1093/nar/gku111 PMC4005661 24510097

[78] Luo Y, Granzhan A, Verga D, Mergny J-L. FRET-MC: A fluorescence melting competition assay for studying G4 structures in vitro. Biopolymers. 2021;112(4): e23415. 33368198 10.1002/bip.23415

[79] Gabelica V. Native Mass Spectrometry and Nucleic Acid G-Quadruplex Biophysics: Advancing Hand in Hand. Acc Chem Res. 2021;54(19):3691–9. 34546031 10.1021/acs.accounts.1c00396

[80] Collie GW, Parkinson GN, Neidle S, Rosu F, De Pauw E, Gabelica V. Electrospray Mass Spectrometry of Telomeric RNA (TERRA) Reveals the Formation of Stable Multimeric G-Quadruplex Structures. J Am Chem Soc. 2010;132(27):9328–34. 20565109 10.1021/ja100345z

[81] Rota Sperti F, Charbonnier T, Lejault P, Zell J, Bernhard C, Valverde IE, et al. Biomimetic, Smart, and Multivalent Ligands for G-Quadruplex Isolation and Bioorthogonal Imaging. ACS Chem Biol. 2021;16(5):905–14. 33914525 10.1021/acschembio.1c00111

[82] Narvi E, Vaparanta K, Karrila A, Chakroborty D, Knuutila S, Pulliainen A, et al. Different responses of colorectal cancer cells to alternative sequences of cetuximab and oxaliplatin. Sci Rep. 2018;8(1):16579. 30410004 10.1038/s41598-018-34938-y PMC6224565

[83] Bugaut A, Balasubramanian S. 5 ’-UTR RNA G-quadruplexes: translation regulation and targeting. Nucleic Acids Res. 2012;40(11):4727–41. 22351747 10.1093/nar/gks068 PMC3367173

[84] Figueiredo J, Mergny J-L, Cruz C. G-quadruplex ligands in cancer therapy: Progress, challenges, and clinical perspectives. Life Sci. 2024:122481. 10.1016/j.lfs.2024.122481 38301873

[85] Kosiol N, Juranek S, Brossart P, Heine A, Paeschke K. G-quadruplexes: A promising target for cancer therapy. Mol Cancer. 2021;20(20):40. 33632214 10.1186/s12943-021-01328-4 PMC7905668

[86] Lejault P, Mitteaux J, Rota Sperti F, Monchaud D. How to untie G-quadruplex knots and why? Cell Chem Biol. 2021;28:436–55. 33596431 10.1016/j.chembiol.2021.01.015

[87] Fracchioni G, Vailati S, Grazioli M, Pirota V. Structural Unfolding of G-Quadruplexes: From Small Molecules to Antisense Strategies. Molecules. 2024;29(15):3488. 39124893 10.3390/molecules29153488 PMC11314335

[88] Zell J, Rota Sperti F, Britton S, Monchaud D. DNA folds threaten genetic stability and can be leveraged for chemotherapy. RSC Chem Biol. 2021;2:47–76. 35340894 10.1039/d0cb00151a PMC8885165

[89] De Magis A, Limmer M, Mudiyam V, Monchaud D, Juranek S, Paeschke K. UV-induced G4 DNA structures recruit ZRF1 which prevents UV-induced senescence. Nat Commun. 2023;14:6705. 37872164 10.1038/s41467-023-42494-x PMC10593929

[90] Alexandre D, Polido J, Miranda A, Hudson RHE, Monchaud D, Baptista PV, et al. Cellular modulation of a G-quadruplex structure found in the lung cancer-related microRNA-3196. Int J Biol Macromol. 2025:145263. 10.1016/j.ijbiomac.2025.145263 40527376

[91] Nicoletto G, Terreri M, Maurizio I, Ruggiero E, Cernilogar Filippo M, Vaine Christine A, et al. G-quadruplexes in an SVA retrotransposon cause aberrant TAF1 gene expression in X-linked dystonia parkinsonism. Nucleic Acids Res. 2024;52:11571–86. 39287133 10.1093/nar/gkae797 PMC12053379

[92] Vichai V, Kirtikara K. Sulforhodamine B colorimetric assay for cytotoxicity screening. Nat Protoc. 2006;1(3):1112. 17406391 10.1038/nprot.2006.179

[93] Zhou G, Liu X, Li Y, Xu S, Ma C, Wu X, et al. Telomere targeting with a novel G-quadruplex-interactive ligand BRACO-19 induces T-loop disassembly and telomerase displacement in human glioblastoma cells. Oncotarget. 2016;7(12):14925. 26908447 10.18632/oncotarget.7483 PMC4924762

[94] Neidle S. Quadruplex nucleic acids as targets for anticancer therapeutics. Nat Rev Chem. 2017;1:0041.

[95] Wu T, Du Y. LncRNAs: From basic research to medical application. Int J Biol Sci. 2017;13(3):295–307. 28367094 10.7150/ijbs.16968 PMC5370437

[96] Lejault P, Moruno-Manchon JF, Vemu SM, Honarpisheh P, Zhu L, Kim N, et al. Regulation of autophagy by DNA G-quadruplexes. Autophagy. 2020;16:2252–9. 32420812 10.1080/15548627.2020.1769991 PMC7751500

[97] Moruno-Manchon JF, Lejault P, Wang Y, McCauley B, Honarpisheh P, Scheihing DAM, et al. Small-molecule G-quadruplex stabilizers reveal a novel pathway of autophagy regulation in neurons. eLife. 2020;9:e52283. 10.7554/eLife.52283 PMC7012600 32043463

[98] Tabor N, Ngwa C, Mitteaux J, Meyer MD, Moruno-Manchon JF, Zhu L, et al. Differential responses of neurons, astrocytes, and microglia to G-quadruplex stabilization. Aging. 2021;13(12):15917–41. 34139671 10.18632/aging.203222 PMC8266374

[99] Escarcega RD, Patil AA, Moruno-Manchon JF, Urayama A, Marrelli SP, Kim N, et al. Pirh2-dependent DNA damage in neurons induced by the G-quadruplex ligand pyridostatin. J Biol Chem. 2023;299(10): 105157. 37579947 10.1016/j.jbc.2023.105157 PMC10534229

[100] Tauchi T, Shin-ya K, Sashida G, Sumi M, Nakajima A, Shimamoto T, et al. Activity of a novel G-quadruplex-interactive telomerase inhibitor, telomestatin (SOT-095), against human leukemia cells: involvement of ATM-dependent DNA damage response pathways. Oncogene. 2003;22(34):5338–47. 12917635 10.1038/sj.onc.1206833

[101] Ye J, Coulouris G, Zaretskaya I, Cutcutache I, Rozen S, Madden TL. Primer-BLAST: a tool to design target-specific primers for polymerase chain reaction. BMC Bioinformatics. 2012;13:1–11. 22708584 10.1186/1471-2105-13-134 PMC3412702

[102] Marchand A, Gabelica V. Native electrospray mass spectrometry of DNA G-quadruplexes in potassium solution. J Am Soc Mass Spectrom. 2014;25(7):1146–54. 24781455 10.1007/s13361-014-0890-3 PMC4055847

[103] Konermann L. Addressing a Common Misconception: Ammonium Acetate as Neutral pH “Buffer” for Native Electrospray Mass Spectrometry. J Am Soc Mass Spectrom. 2017;28(9):1827–35. 28710594 10.1007/s13361-017-1739-3

