# Supplementary material for: Pharmacomodulation of G-quadruplexes in long non-coding RNAs dysregulated in colorectal cancer
Source: BMC Biol. 2025 Aug 8;23:249. doi: 10.1186/s12915-025-02322-8 (PMC12333089; doi:10.1186/s12915-025-02322-8)
Supplement: Supplementary file 1 — Additional file 1: details about 1-the selection of lncRNA using QGRS mapper [61] and G4Hunter [63], 2- the preparation of the oligonucleotides notably primers using primer-BLAST [101], 3- the reagents used in this study, 4- the cell lines and related culture protocols, 5- the ESI–MS protocol [102, 103] and 6- the SRB method [92]. Figure S1: Identification of Putative Quadruplex-forming Sequences (PQS) in long non-coding RNAs (lncRNAs) dysregulated in Colorectal cancer (CRC). Figure S2: Expression of LINC01589, MELTF-AS1, and UXT-AS1 lncRNAs in different cancer cell lines. Figure S3: CD and UV spectra, along with thermal difference signature (TDS) [65] calculated for LINC01589, MELTF-AS1, and UXT-AS1 lncRNAs. Figure S4: in vitro formation of stable G4s in LINC01589, MELTF-AS1, and UXT-AS1 lncRNAs assessed via N-TASQ fluorescence enhancement assay. Figure S5: fluorescence quenching assay (FQA) [74] to determine the affinity of G4-interacting molecules for either DNA G4 (Cy5-Myc) or RNA G4 (Cy5-NRAS). Figure S6: CD-melting experiments performed lncRNA G4s in the presence of ligands. Figure S7: UV-melting experiments performed with lncRNA G4s in the presence of ligands. Figure S8: Fluorescence titrations were performed with RNA G4s and ThT. Figure S9. ESI–MS analysis of folded synthetic RNA G4s. Figure S10: Cytotoxicity of PhpC and BRACO-19 in HT-29 cells using the SRB test. Figure S11. G4RP-RT-qPCR results [57, 73] obtained with BRACO19 and PhpC on LINC01589, MELTF-AS1, and UXT-AS1 lncRNA G4s in HT-29 cells. [file 12915_2025_2322_MOESM1_ESM.pdf]

SUPPLEMENTAL INFORMATION

Pharmacomodulation of G-quadruplexes in long non-coding RNAs dysregulated in colorectal cancer

Shubham Sharma[1], Jérémie Mitteaux[2], Angélique Pipier[2], Marc Pirrotta[2], Marie-José Penouilh[2], David Monchaud[2,*] and Bhaskar Datta[1, 3,*]

[1]Department of Biological Sciences and Engineering, Indian Institute of Technology Gandhinagar, Gandhinagar, Gujarat, 382055, India. [2]Institut de Chimie Moléculaire de l'Université de Bourgogne (ICMUB), CNRS UMR6302, Université Bourgogne Europe (UBE) Dijon, 21000, France. [3]Department of Chemistry, Indian Institute of Technology Gandhinagar, Gandhinagar, Gujarat, 382055, India [*]Correspondence to david.monchaud@cnrs.fr and bdatta@iitgn.ac.in

LncRNA selection criteria for *in vitro* investigation. The dataset generated by *in silico* G4-prediction of PQS in lncRNAs using QGRS mapper and G4Hunter was segmented based on their anticipated G4 types (2G, 3G, and 4G) (Figure S1A; Table S1). The segmented data set was then sequentially filtered to obtain three pools of lncRNAs fulfilling the following conditions:

Pool A [2G: lncRNAs with ≤5 2G-PQS; one transcript variant; no 3G- or 4G-PQS; PQS from G4Hunter should be similar to/part of PQS from QGRS mapper];

Pool B [3G: lncRNAs with one 3G-PQS; one transcript variant; PQS G-Score ≥ 60 (QGRS mapper); no 2G- or 4G-PQS; PQS from G4Hunter should be similar to/part of PQS from QGRS mapper];

Pool C [4G: lncRNAs with one 4G-PQS; one transcript variant; no 2G- or 3G-PQS; PQS from G4Hunter should be similar to/part of PQS from QGRS mapper].

The selection approach aimed to include one representative lncRNA from each of the above-mentioned pools, to ensure a comprehensive analyses of different anticipated G4 types. The high-scoring PQS were observed in *LINC01589*, *MELTF-AS1*, and *UXT-AS1* lncRNAs with the potential to fold into a 2G-G4 (2G-PQS), 3G-G4 (3G-PQS) and 4G-G4 (4G-PQS), respectively, as predicted by the QGRS mapper. In *LINC01589*, while all 3 PQS were found to be 2G-PQS, 1 of them displayed the highest G-score of 35, and no 3G- or 4G-PQS were observed. Among 8 PQS in *MELTF-AS1*, 1 3G-PQS displayed the highest G-score of 64, while no 4G-PQS was observed. Out of 4 PQS in *UXT-AS1*, 1 4G-PQS displayed the highest G-score of 90, while no 3G-PQS was observed (Figure S1B; Table S1). Interestingly, the PQS displaying high G-scores in the QGRS mapper across these lncRNAs were also identified by G4Hunter as the PQS possessing a high propensity to form G4s based on suitable high G4Hunter Scores (Figure S1C; Table S1). The high-scoring 2G-, 3G-, and 4G-

PQS of *LINC01589*, *MELTF-AS1*, and *UXT-AS1* were then selected for *in vitro* investigation of their formation (Figure S1B,C).

Oligonucleotides. RNA oligonucleotides corresponding to the Putative G-quadruplex-forming Sequences (PQS) of selected lncRNAs along with the FAM-21-TAMRA (F21T) DNA oligonucleotide were sourced from Kaneka Eurogentec S.A., Belgium. DNA primers for Reverse Transcription-quantitative Polymerase Chain Reaction (RT-qPCR) were designed using Primer-BLAST (NCBI, USA) and acquired from Kaneka Eurogentec S.A., Belgium. The oligonucleotides were reconstituted in the nuclease-free water to a final concentration of 100–500 µM and stored at -20 °C until further use. Details regarding the sequences of the DNA and RNA oligonucleotide mentioned above are accessible in **Table S2-S3**.

Reagents. Dulbecco's Modified Eagle Medium (Catalog no. L0104 and 11965092) and Fetal bovine serum (FBS) (Catalog no. S1810 and 10270106) were obtained from Dominique DUTSCHER SAS, France and Thermo Fischer Scientific India Pvt. Ltd., Mumbai, India. RPMI 1640 Medium (Catalog no. 21870076), Leibovitz's L-15 Medium (Catalog no. 41300039), GlutaMAX™ (Catalog no. 35050061), L-Glutamine (Catalog no. 25030149) and Penicillin-Streptomycin (Catalog no. 15140122) were acquired from Thermo-Fischer Scientific Inc., Fisher Scientific SAS, France and Thermo Fischer Scientific India Pvt. Ltd., Mumbai, India. TRIzol™ Reagent (Catalog no. 15596018), SuperScript™ III Reverse Transcriptase (Catalog no. 18080093), RNaseOUT™ Recombinant Ribonuclease Inhibitor (Catalog no. 10777019), Random Hexamers (Catalog no. N8080127) and Oligo(dT)$_{20}$ Primer (Catalog no. 18418020) were procured from Thermo-Fischer Scientific Inc., Fisher Scientific SAS, France. RNA Clean & Concentrator-5 kit (DNase Included) (Catalog no. R1013) was obtained from Zymo Research Europe, Germany. iTaq Universal SYBR Green Supermix (Catalog no. 1725121) was procured from Bio-Rad, France. Biotin (Catalog no. B4501) was acquired from Sigma Aldrich Chimie S.a.r.l, France. Streptavidin MagneSphere® Paramagnetic Particles (Catalog no. Z5481) were procured from Promega France, France. All the reagents were prepared following the manufacturer's protocol and stored in the recommended conditions.

Cell lines. Colorectal cancer cell lines, including COLO205, COLO320DM, HCT-15, HT-29, HCT-116 and SW620, were procured from the National Cell Repository for cell lines at the National Centre for Cell Science (NCCS), Pune, India. Cell lines from breast cancer [HCC-1954 (CRL-2338), MDA-MB-231 (HTB-26), MCF7 (HTB-22)], prostate cancer [PC3 (CRL-1435)] and cervical cancer [HeLa (CCL-2)] were acquired from the American Type Culture Collection (ATCC), USA. HT-29, HCT-116, HeLa and MCF7 were cultured in Dulbecco's Modified Eagle Medium (DMEM), while

COLO205, COLO320DM, HCT-15, HCC-1954, MDA-MB-231 and PC3 were cultured in RPMI 1640 Medium. SW620 was cultured in Leibovitz's L-15 Medium. All cell lines were supplemented with 10% (v/v) Fetal Bovine Serum and 1% (v/v) Penicillin-Streptomycin. Additionally, cell lines cultured in RPMI 1640 medium were supplemented with 1% (v/v) GlutaMAX™ or L-Glutamine. All the cell lines were maintained at 37 ºC in the presence of 5% CO_2, except SW620, which was maintained in the absence of 5% CO_2.

Preparation of RNAs. The synthetic RNAs corresponding to the high-scoring PQS within lncRNAs (**Table S3**) were reconstituted in the nuclease-free water to a final concentration of 500 µM and stored at -20 °C until further use. The RNAs (1 µM) were subjected to heating at 95 °C for 5 minutes in the presence of a folding buffer: 10 mM Tris-HCl (pH 7.5) and 0.1 mM EDTA (pH 8.0), supplemented with 10 mM KCl. The RNAs were then gradually cooled to room temperature to facilitate the formation of the G4s.

Hydrophilic Interaction Liquid Chromatography (HILIC) coupled with Electrospray Ionization Mass Spectrometry (ESI-MS). RNAs (10 µM) were folded in 100 mM Ammonium Acetate solution (pH 7.0) to avoid incompatibility with the native electrospray ionization on the usage of non-volatile solutions, and minimize any pH-induced effects. Methanol was added to the folded RNAs to a working concentration of 20%. The prepared sample was introduced into the Vanquish HPLC System (Thermo-Fischer Scientific Inc., Fisher Scientific SAS, France) with Luna 3 µM HILIC column (Catalog no. 00D-4449-B0, 100 X 2 mm, Phenomenex, France) and subjected to a gradient (10/90 to 40/60) of mobile phase: 20 mM Ammonium Formate (pH 3.2) and 100% Acetonitrile, for 10 minutes, to generate the chromatogram. The UV-Vis detector was used at 260 nm to determine the RNA content with respect to the HILIC chromatogram. Samples were subsequently injected into the Orbitrap Exploris™ 240 ESI-MS (Thermo-Fischer Scientific Inc., Fisher Scientific SAS, France) and analysed in negative mode using the following parameters, flow rate: 500 µl/min; resolution: 120000; mass/charge range: 500 to 6000; scan type: full and RF lens: 100%. The HILIC chromatogram and UV-Vis data were used in conjunction to determine the retention time(s) where the RNAs exhibited their highest relative abundance, with the most significant absorption at 260 nm. ESI-MS spectra were generated for the identified retention time(s), and the m/z peaks with high relative abundances were analysed for their experimental mass.

Sulforhodamine B (SRB) cytotoxicity assay with ligand. HT-29 cells were seeded at $8x10^3$ density in a 96-well tissue culture microtiter plate and cultured at 37 °C in the presence of 5% CO_2 for 24 hours. The cells were treated with increasing concentrations (0–100 µM) of PhpC or BRACO-19 after

24 hours of seeding. After 72 hours of treatment, the cells were fixed with 10% (w/v) TCA and stained with the SRB dye for 30 minutes. Excess SRB dye was effectively removed through a series of thorough washes using a 1% (v/v) acetic acid solution. The SRB dye, bound to the cellular proteins, was solubilized in a 10 mM Tris base solution for optical density (OD) measurement at 530 nm using a CLARIOstar® Plus multimode plate reader (BMG LABTECH SARL, France). The data were recorded in duplicates across three independent studies. HT-29 cell viability at each concentration of PhpC was estimated by calculating the change in the OD at each concentration of PhpC with respect to the OD of negative control (in the absence of PhpC). The mean HT-29 cell viability (%) values were normalized and plotted against the concentration of the ligand used for HT-29 cells using GraphPad Software. The plot was fit using the dose response-inhibition model of non-linear regression, to determine the IC_{50} values for PhpC in HT-29 cells.

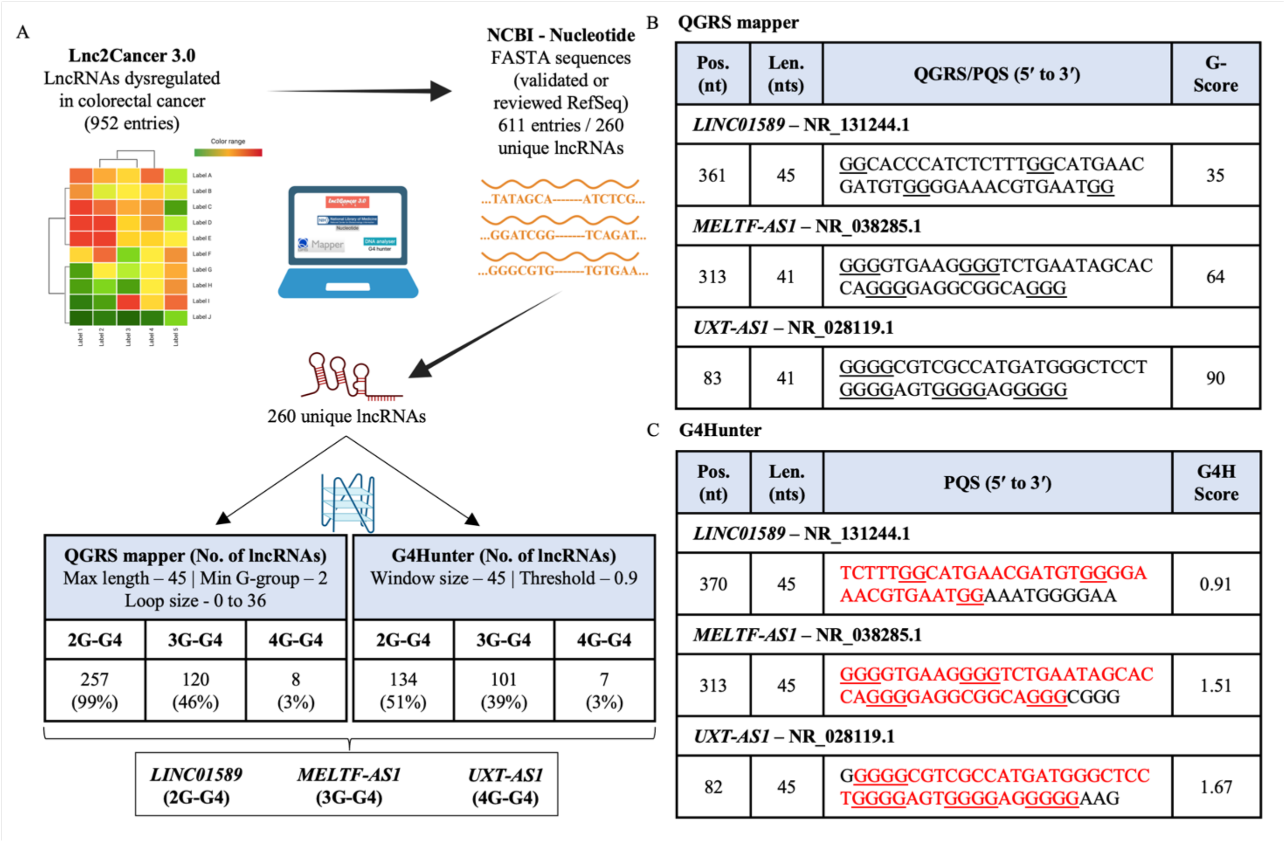

Figure S1. Identification of Putative Quadruplex-forming Sequences (PQS) in long non-coding RNAs (lncRNAs) dysregulated in Colorectal cancer (CRC). A) *In silico* prediction of PQS in lncRNAs dysregulated in CRC using various databases and G4-prediction tools. A list of CRC-dysregulated lncRNAs is obtained from Lnc2Cancer 3.0, and their respective FASTA sequences are obtained from NCBI nucleotide. LncRNA FASTA sequences are used in G4-prediction tools: QGRS mapper and G4Hunter, to obtain PQS. A 2G, 3G, and 4G PQS from *LINC01589*, *MELTF-AS1*, and *UXT-AS1* lncRNAs, respectively, are shortlisted for *in vitro* investigation. See the "*LncRNA selection criteria for in vitro investigation*" section above for details about the *in silico* methodology. B-C) PQS identified from selected lncRNAs using B) QGRS mapper, and C) G4Hunter. Regions of PQS obtained from QGRS mapper overlapping with PQS from G4Hunter are marked in red.

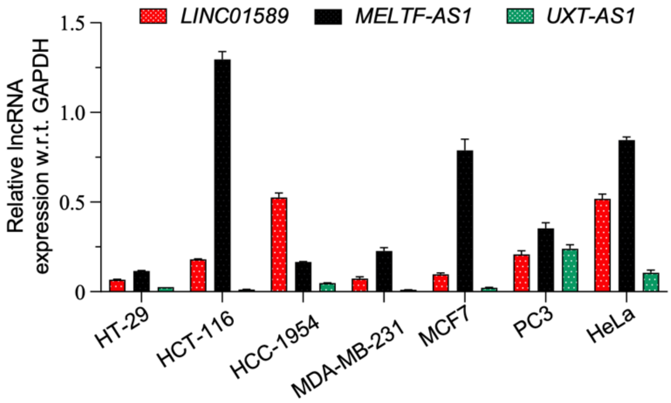

Figure S2. Expression of *LINC01589*, *MELTF-AS1*, and *UXT-AS1* lncRNAs in different cancer cell lines. Reverse Transcription-quantitative Polymerase Chain Reaction (RT-qPCR) with RNAs isolated from CRC (HT-29, HCT-116), breast cancer (HCC-1954, MDA-MB-231, MCF7), prostate cancer (PC3), and cervical cancer (HeLa) cell lines show mean relative lncRNA expression with respect to GAPDH ($2^{-\Delta Ct}$) ± SEM. Data recorded in technical triplicate from three independent studies.

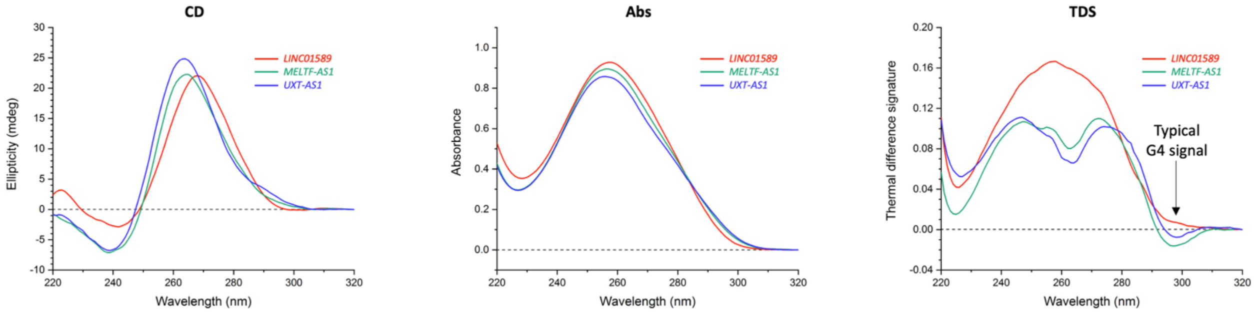

Figure S3. CD and UV spectra recorded in 10 mM lithium cacodylate buffer (pH 7.2) plus 10 mM KCl and 90 mM LiCl with 1 µM lncRNA G4s, along with thermal difference signature (TDS) calculated for *LINC01589*, *MELTF-AS1*, and *UXT-AS1* lncRNAs. Data recorded in technical triplicate from three independent studies; averaged spectra are shown.

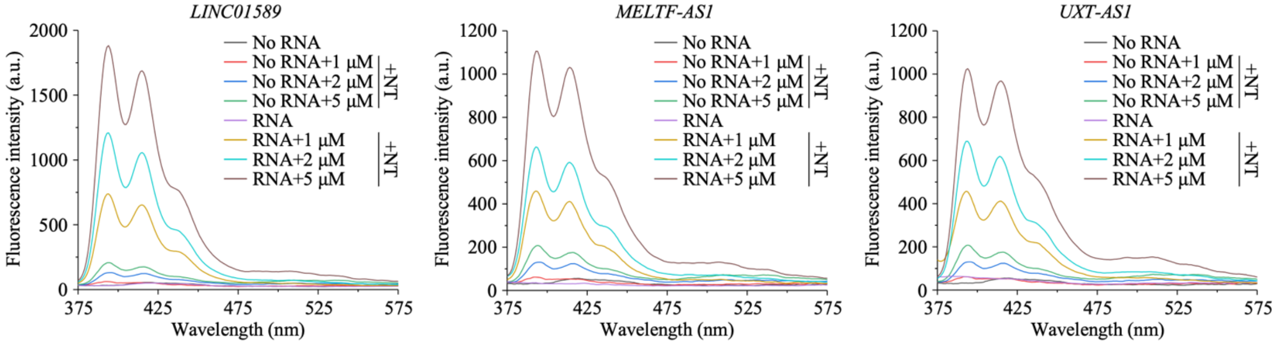

Figure S4. *In vitro* formation of stable G4s in *LINC01589*, *MELTF-AS1*, and *UXT-AS1* lncRNAs. N-TASQ fluorescence enhancement assay of synthetic RNAs (2 µM) folded in 10 mM Tris-HCl (pH 7.5) and 0.1 mM EDTA (pH 8.0), and titrated with N-TASQ (NT, 1 – 5 µM). Increased mean fluorescence emission spectra when excited at 280 nm show G4-formation. Data recorded in technical triplicate from three independent studies; averaged spectra are shown.

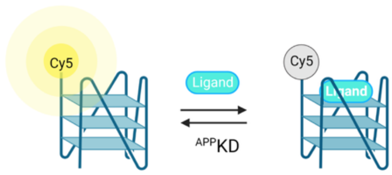

	BRACO-19	PhpC	N-TASQ	BioCyTASQ	MultiTASQ	azMultiTASQ
**Cy5-Myc**	0.37 ± 0.06	> 100	1.00 ± 0.03	1.01 ± 0.11	0.65 ± 0.11	0.53 ± 0.14
**Cy5-NRAS**	0.86 ± 0.26	> 100	0.51 ± 0.01	0.66 ± 0.00	0.50 ± 0.05	0.51 ± 0.02

Figure S5. Standardized assay (fluorescence quenching assay, FQA) to determine the affinity (${}^{app}K_D$, in µM) of G4-interacting molecules for either DNA G4 (Cy5-Myc) or RNA G4 (Cy5-NRAS). Comparison of the results obtained with BRACO-19, PhpC and a series of TASQs (N-TASQ, BioCyTASQ, MultiTASQ and azMultiTASQ). Experiments were performed with 200 nM of labelled oligonucleotide in Tris HCl buffer (50 mM Tris, 150 mM KCl, 0.5% (v/v) triton, pH 7.2) at 25 °C for 1 h, in the presence of ligands (from 6.10^{-3} to 100 µM).

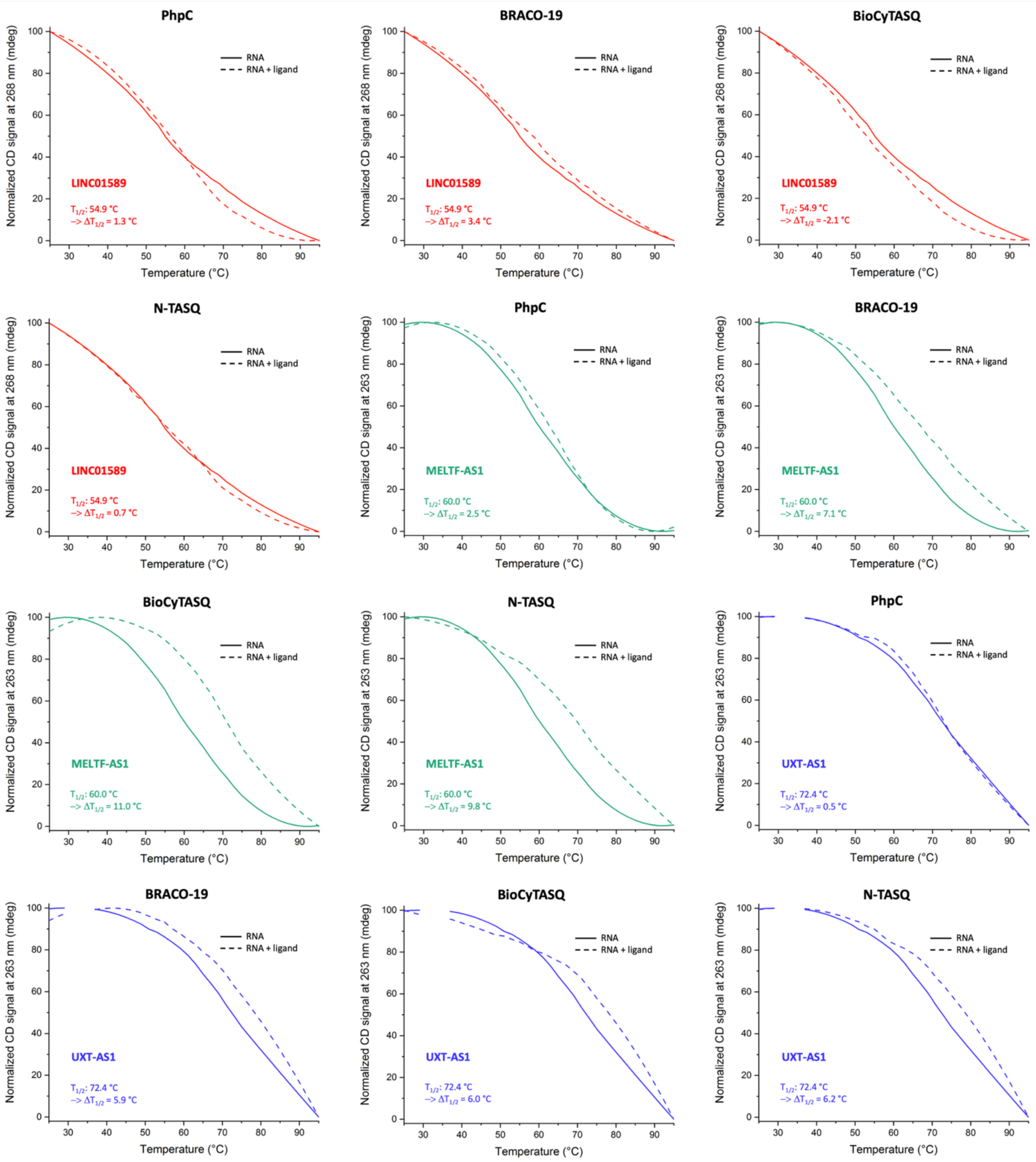

Figure S6. CD-melting experiments performed with 1 µM lncRNA G4s in 10 mM lithium cacodylate buffer (pH 7.2) plus 10 mM KCl and 90 mM LiCl in the presence of 5 µM ligand; melting experiments were conducted upon temperature change (from 25 °C to 95 °C) at 2 °C/min rate. Data recorded in technical triplicate; averaged spectra are shown.

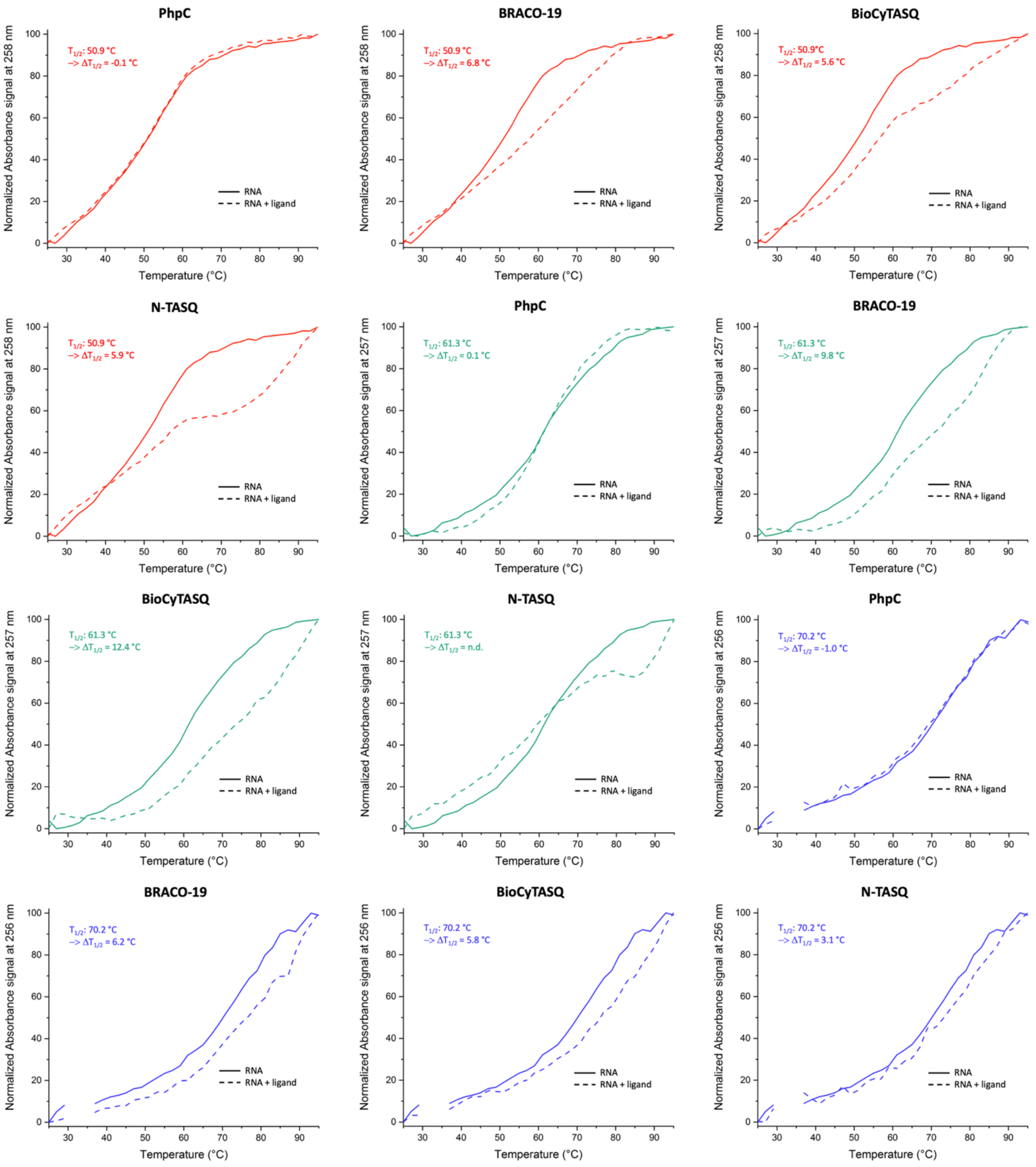

Figure S7. UV-melting experiments performed with 1 µM lncRNA G4s in 10 mM lithium cacodylate buffer (pH 7.2) plus 10 mM KCl and 90 mM LiCl in the presence of 5 µM ligands; melting experiments were conducted upon temperature change (from 25 °C to 95 °C) at 2 °C/min rate. Of note, "n.d." for N-TASQ, which stands for not determined, is related to the multiphasic nature of the curve obtained, which makes it unsuited to quantification. Data recorded in technical triplicate; averaged spectra are shown.

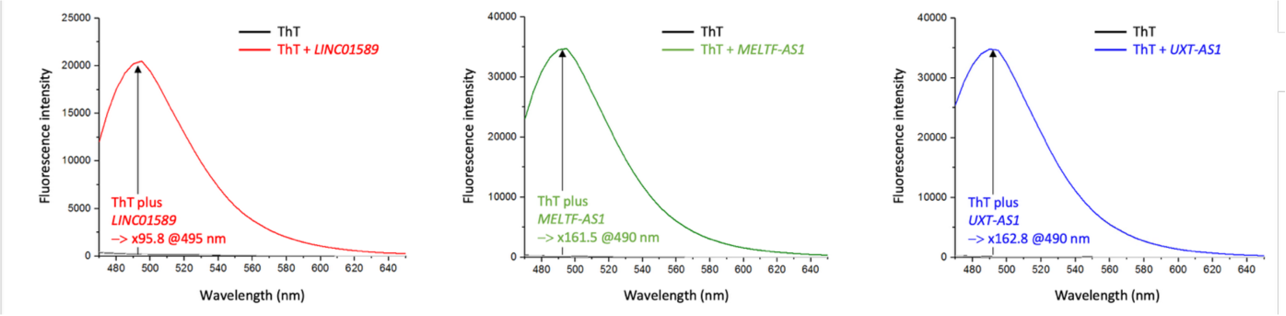

Figure S8. Fluorescence titrations were performed with RNAs (2 µM) and ThT (2 µM); the fluorescence emission (from 470 to 650 nm) was recorded at 25 °C in 10 mM lithium cacodylate buffer (pH 7.2) plus 10 mM KCl and 90 mM LiCl. Data recorded in technical triplicate; averaged spectra are shown.

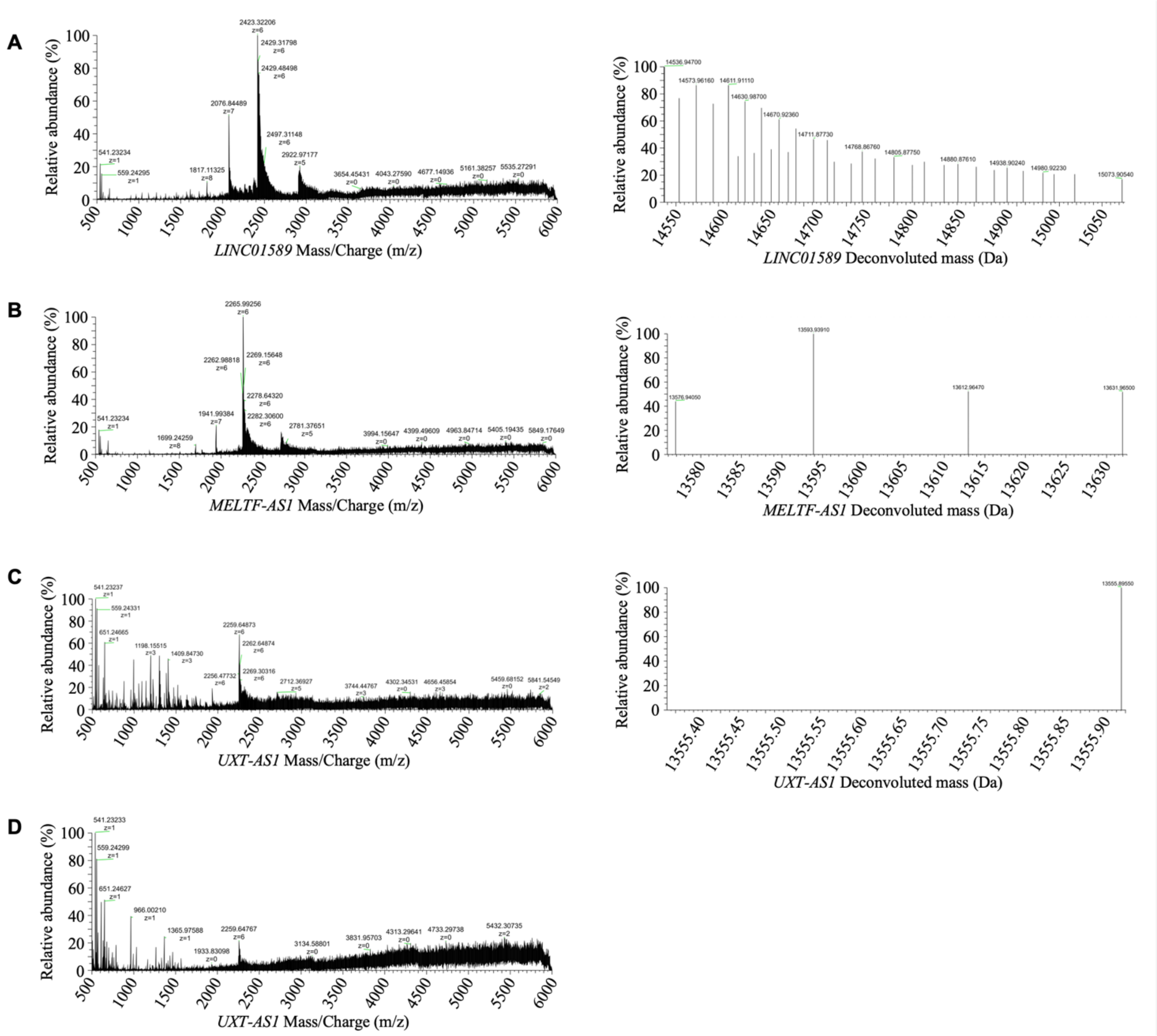

Figure S9. A-C) Electrospray Ionization Mass Spectrometry (ESI-MS) mass/charge (m/z) spectra (left) and deconvoluted spectra (right) of folded synthetic RNAs (10 µM) show highest abundance MS peaks corresponding to the mass of unimolecular or intramolecular G4s. D) ESI-MS m/z spectra of an additional peak of folded synthetic *UXT-AS1* lncRNA (10 µM) with high abundance and significance absorbance at 260 nm in the HILIC chromatogram and UV-Vis data, respectively, shows an MS peak corresponding to the mass of unimolecular or intramolecular G4.

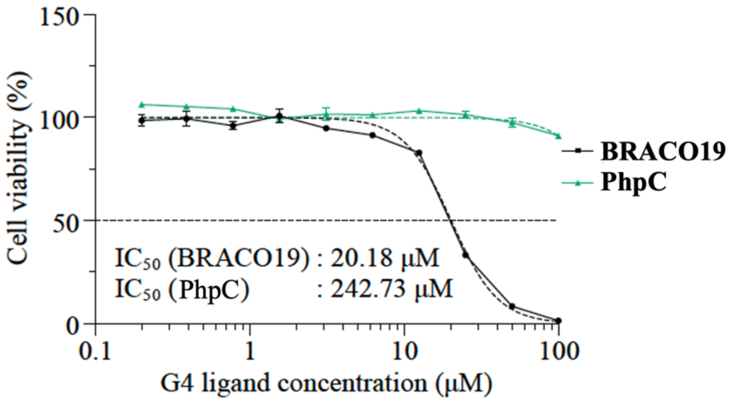

Figure S10. Cytotoxicity of PhpC and BRACO-19 in HT-29 cells using Sulforhodamine B (SRB) dye. Cells are treated with PhpC (0 – 100 µM) for 72 hours. Mean ± SD of normalized cell viability at different PhpC concentrations indicates the half-maximal inhibitory concentration (IC_{50}) of PhpC. Data recorded in technical triplicate from three independent studies.

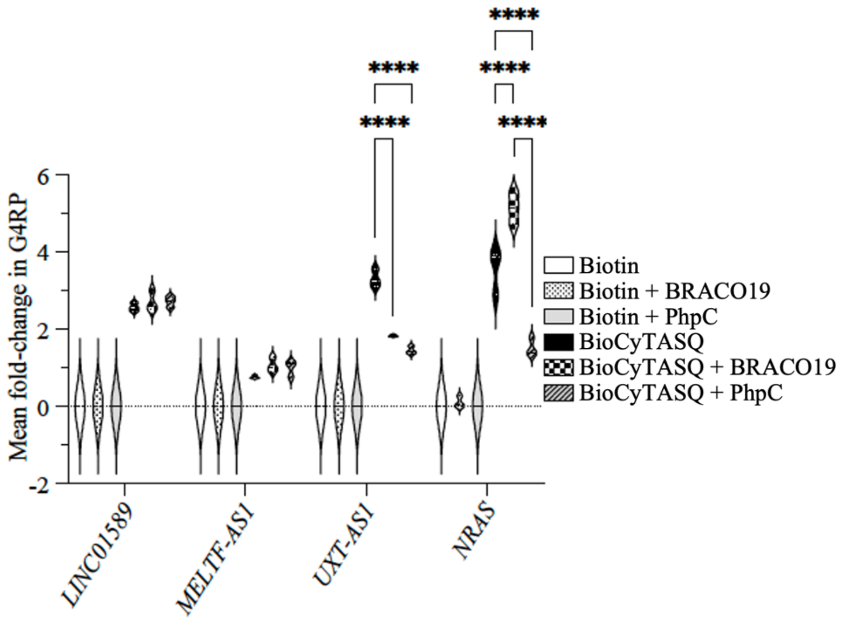

Figure S11. G4RP-RT-qPCR results obtained with BRACO19 (10 µM) and PhpC (90 µM) on LINC01589, MELTF-AS1, and UXT-AS1 lncRNA G4s in HT-29 cells. Mean ± SD fold-change in the level of RNAs [5 *{2(Mean Ct input – Ct G4RP or biotin)}] indicate the effect of ligands on the RNA G4s. The results are collected from triplicates (n = 3) across three independent studies (n = 3). Two-way ANOVA was applied for the statistical analyses, with *: $P \leq 0.05$, **: $P \leq 0.01$, ***: $P \leq 0.001$, ****: $P \leq 0.0001$; non-significant *P*-values are not represented.
